# Ovatodiolide Targets ****β****-Catenin Signaling in Suppressing Tumorigenesis and Overcoming Drug Resistance in Renal Cell Carcinoma

**DOI:** 10.1155/2013/161628

**Published:** 2013-05-26

**Authors:** Jar-Yi Ho, Ren-Jun Hsu, Chieh-Lin Wu, Wen-Liang Chang, Tai-Lung Cha, Dah-Shyong Yu, Cheng-Ping Yu

**Affiliations:** ^1^Graduate Institute of Life Sciences, National Defense Medical Center, No. 161, Sec. 6, Minquan E. Road, Neihu District, Taipei 114, Taiwan; ^2^Department of Pathology and Graduate Institute of Pathology and Parasitology, Tri-Service General Hospital, National Defense Medical Center, No. 161, Sec. 6, Minquan E. Road, Neihu District, Taipei 114, Taiwan; ^3^Biobank Management Center of Tri-Service General Hospital, National Defense Medical Center, No. 161, Sec. 6, Minquan E. Road, Neihu District, Taipei 114, Taiwan; ^4^School of Pharmacy, National Defense Medical Center, No. 161, Sec. 6, Minquan E. Road, Neihu District, Taipei 114, Taiwan; ^5^Division of Urology, Tri-Service General Hospital, National Defense Medical Center, No. 161, Sec. 6, Minquan E. Road, Neihu District, Taipei 114, Taiwan

## Abstract

Dysregulated **β**-catenin signaling is intricately involved in renal cell carcinoma (RCC) carcinogenesis and progression. Determining potential **β**-catenin signaling inhibitors would be helpful in ameliorating drug resistance in advanced or metastatic RCC. Screening for **β**-catenin signaling inhibitors involved *in silico* inquiry of the PubChem Bioactivity database followed by TCF/LEF reporter assay. The biological effects of ovatodiolide were evaluated in 4 RCC cell lines *in vitro* and 2 RCC cell lines in a mouse xenograft model. The synergistic effects of ovatodiolide and sorafenib or sunitinib were examined in 2 TKI-resistant RCC cell lines. Ovatodiolide, a pure compound of *Anisomeles indica*, inhibited **β**-catenin signaling and reduced RCC cell viability, survival, migration/invasion, and *in vitro* cell or *in vivo* mouse tumorigenicity. Cytotoxicity was significantly reduced in a normal kidney epithelial cell line with the treatment. Ovatodiolide reduced phosphorylated **β**-catenin (S552) that inhibited **β**-catenin nuclear translocation. Moreover, ovatodiolide decreased **β**-catenin stability and impaired the association of **β**-catenin and transcription factor 4. Ovatodiolide combined with sorafenib or sunitinib overcame drug resistance in TKI-resistant RCC cells. Ovatodiolide may be a potent **β**-catenin signaling inhibitor, with synergistic effects with sorafenib or sunitinib, and therefore, a useful candidate for improving RCC therapy.

## 1. Introduction

Renal cell carcinoma (RCC) is the most lethal genitourinary cancer, and the worldwide incidence and mortality rates of RCC have increased annually. In 2008, the incidence was 4/100,000 and the mortality 1.6/100,000 people worldwide. The incidence is 3.2/100,000 and mortality 1.7/100,000 people in Taiwan [1, 2]. Most advanced RCC is highly refractory to chemotherapy and radiation therapy and has reduced the 5-year survival to 0–20% [3, 4].

Six targeted agents for treating advanced or metastatic RCC are now approved and in clinical use. Three are tyrosine kinase inhibitors (TKIs), including sunitinib, pazopanib, and sorafenib. TKIs could improve the overall survival of RCC patients [5, 6]. Other agents include an antivascular endothelial growth factor, monoclonal antibody bevacizumab, and 2 mammalian targets of rapamycin inhibitors, temsirolimus and everolimus [7–12]. However, limited efficacy has been reported for these drugs [5], and more potent compounds that target specific signaling pathways of RCC pathogenesis are needed to improve the high rate of refractory disease.

The *β*-catenin signaling pathway is intricately involved in RCC carcinogenesis and progression. Several **β**-catenin signaling components have been examined in RCC recently, and **β**-catenin signaling may be constitutively active in RCC [13, 14]. Aberrant activation of **β**-catenin signaling is involved in RCC carcinogenesis and progression and in the overexpression or overactivation of **β**-catenin [13, 14] and oncogenic WNT10A ligand [15] as well as genetic or epigenetic dysregulation of WNT antagonists [16, 17]. *β*-Catenin overexpression in RCC was associated with increased incidence and poor prognosis [16, 18–20]. The investigation of canonical **β**-catenin signaling and RCC has focused on genetic and epigenetic changes of WNT antagonistic genes [14]. For instance, Dickkopf 2 (DKK2) rs17037102 and DKK3 rs1472189 polymorphisms were found associated with RCC prognosis [21]. The epigenetic silencing of WNT antagonistic genes, including secreted Frizzled-related proteins, DKKs, and WNT inhibitory factor 1, was highly correlated with poor RCC prognosis [14]. Some biologic and small-molecule inhibitors of **β**-catenin signaling have been used to develop novel cancer therapeutic agents [22, 23] but scantily for RCC treatment and chemoresistance [24]. To our knowledge, only two pharmaceutical **β**-catenin inhibitors, RX-8243 [25] and BC2059 [26], had been reported to reduce cell proliferation in RCC cell lines.

High-throughput screening to identify modulators of molecular targets has been used with crude extracts or pure compounds isolated from Chinese herbs. The technique can comprehensively delineate relationships among compound structures and biological activities for biological and clinical relevance in specific diseases. Here, we used an easily accessed database for direct cytotoxic screening of compounds that might be effective in RCC. There are ~25% metastatic RCC patients appeared no clinical benefit of TKIs or responded to TKIs initially but go onto disease progression after a median of 5–11 months [27–32]. Discovery of specific inhibitors of other critical signaling pathways would help improve RCC therapy. In this study, we screened for inhibitors of **β**-catenin signaling and evaluated the biological effects of the revealed ovatodiolide in 4 RCC cell lines *in vitro* and 2 RCC cell lines in a mouse xenograft model. We also examined the synergistic effects of ovatodiolide and sorafenib or sunitinib in 2 TKI-resistant RCC cell lines.

## 2. Material and Methods

### 2.1. Cell Lines

The RCC lines 786-O, Caki-1, ACHN, and A498, the nontumorigenic human kidney epithelial cell line HK-2, and the human embryonic kidney cell line HEK293T were obtained from the Bioresource Collection and Research Center (Taiwan). 786-O, Caki-1, and ACHN cell lines were maintained in RPMI-1640 and A498, and HEK293T cells were maintained in Dulbecco's Modified Eagle medium (DMEM), all with 10% fetal bovine serum, 1 *μ*g/mL penicillin, and 1 *μ*g/mL streptomycin (Invitrogen). HK293T cells were grown in keratinocyte serum-free medium supplemented with 50 ng/mL bovine pituitary extract and 5 ng/mL epidermal growth factor. All cells were maintained at 37°C in a 5% CO_2_ atmosphere. To obtain drug-resistant RCC cell lines, 786-O and ACHN were cultured with 5 *μ*M sorafenib or 5 *μ*M sunitinib malate (Santa Cruz Biotechnology) and fresh complete RPMI-1640 was replaced every 3 days. Cells were cultured for 6 months. The parental controls were performed on 786-O and ACHN cells with similar passage numbers with the only difference being the presence of sorafenib or sunitinib malate in the media.

### 2.2. Compound Screening

All compounds for screening were offered by Professor Wen-Liang Chang. After disregarding of the redundant compounds, a total number of 21 pure compounds from *Citrus reticulata* Blanco, 16 from *Hibiscus syriacus* L., and 23 from *Anisomeles indica* L. were selected. In brief, the dried and powdered fruit skins of *C. reticulata*, root barks of *H. syriacus,* or stems of *A. indica* (1.0 kg/each) were extracted sequentially with acetone (5 L, 3 times), methanol (5 L, 3 times), 5 L of ethanol (95%, 60%, and 20%), and water (2 L) under reflux for 2 h. The crude extracts were then defatted with n-hexane, partitioned with chloroform and n-butanol, and chromatographed on a silica gel column by eluting with n-hexane/ethyl acetate gradient, with increasing polarity. Ovatodiolide was prepared as described previously and confirmed by high-performance liquid chromatography (HPLC) (column: RP C18e4.6× 250 mm, 5 *μ*mm (Merck)). The mobile phase consisted of acetonitrile and 0.1% trifluoroacetic acid (TFA) in water, 64:36 (UV detection at 265 nm) [33]. HPLC revealed the purity of compounds to be ~95% pure. Representative HPLC chromatogram of ovatodiolide was shown in Figure S1E in supplementary Matrial available online at http://dx.doi.org/10.1155/2013/161628. We evaluated the cytotoxic potential of each compound. First, *in silico* drug screening involved the use of the PubChem BioActivity database to select each *Active* outcome in any *BioAssay* for human tumor cell growth inhibition or antiproliferative activity, *in vivo *antitumor or anticancer activity, induction of apoptosis, or cytotoxicity (summarized in Table S1). We selected 5 pure compounds for *C. reticulata* Blanco, 4 for *H. syriacus* L., and 2 for *A. indica* L. Second, we used transcription factor/lymphoid enhancer factor (TCF/LEF) reporter assay with these 11 compounds to compare repression of **β**-catenin signal transduction. Psoralen, an abundant pure compound of *Psoralea corylifolia* L., was used as a **β**-catenin signaling control [34]. After 24 hr of transfection with TOPFlash or FOPFlash plasmids, cells were treated with each compound (20, 40 *μ*M) for an additional 24 hr and luciferase activities were measured to evaluate the inhibitory effects of compounds on endogenous **β**-catenin signaling (Table S1). Dimethyl sulfoxide (DMSO) stock solution was kept at −20°C and freshly diluted to the desired concentrations with cell culture medium immediately before use. The final concentration of DMSO in culture medium was 0.1%.

### 2.3. Luciferase Reporter Assay

To detect the activity of **β**-catenin signal transduction, we used the TCF/LEF reporter assay with luciferase reporter plasmids (Super 8x TOPFlash with the wild-type TCF binding sites; Super 8X FOPflash with the mutant-type TCF binding sites (Addgene clone M50, M51)). In addition, pGL3-NFAT luciferase (Addgene 17870), CRE-Luc, and NF*κ*B reporter plasmids (Qiagen) were used to compare the regulatory effects of ovatodiolide in NF-AT- or cAMP-response-element-(CRE-) regulated promoters. The pGL4.71 renilla luciferase vector (Promega) was cotransfected in a 1/40 molar ratio to normalize transfection efficiency with Lipofectamine 2000 (Invitrogen). After 24 hr of transfection, cells were exposed for 24 hr to DMSO or 20 *μ*M ovatodiolide with recombinant human WNT3a (rhWNT3a, 25 ng/mL) or LiCl (20 mM) for TOPFlash, ionomycin (1 *μ*g/mL) for NF-AT luc, forskolin (10 *μ*M) for CRE luc, and tumor necrosis factor *α* (TNF*α*; 10 ng/mL) for NF*κ*B luc activity controls. Assay of luciferase activity at 48 hr involved use of a Dual-Luciferase reporter assay system (Promega). All experiments were performed in triplicate.

### 2.4. RNA Preparation and Quantitative Real-Time PCR

RNA was isolated from treated cells by the use of TRIzol (Invitrogen). RNA samples were treated with RQ1 RNase-free DNase (Promega) to remove any genomic contamination. In all, 5 *μ*g treated RNA sample was used for reverse transcription with SuperScript III (Invitrogen). Quantitative real-time PCR involved the StepOne Real-Time PCR System (Applied Biosystems) with the GM SYBR qPCR Mix Kit (GeneMark) and GAPDH was used as an internal control. Besides analysis of the melting curve, real-time PCR products were analyzed by gel electrophoresis to confirm single PCR products. Primer sets are as in Table S2 [35].

### 2.5. Western Blot Analysis

Treated cells were washed twice with phosphate buffered saline (PBS), then lysed in 200 *μ*L RIPA lysis buffer (Millipore, 50 mM Tris-HCl, pH 7.4, 150 mM NaCl, 1 mM EDTA, 1% Triton X-100, 1% sodium deoxycholate, and 0.1% SDS) containing 2x protease inhibitor (Roche). An amount of 20 *μ*g protein from the supernatant was loaded on SDS polyacrylamide gels and then underwent Western blot analysis to detect the protein level of indicated genes (antibodies are listed in Table S3). For evaluating ovatodiolide specificity, we compared active **β**-catenin (p*-*β**-catenin [S552]) and its downstream genes (c-myc, cyclin D1, and survivin) and other WNT molecules (TCF4, low-density lipoprotein (LDL) receptor-related proteins 5 and 6 (LRP5/6), active LRP5/6 (p-LRP5/6), Axin1, and dishevelled). For apoptotic effects, we compared caspase 3, 8, 9, poly (ADP-ribose) polymerase [PARP] and their cleaved forms, apoptotic proteins Bax, Bid, and PUMA, and antiapoptotic proteins Bcl-2, Bcl-xL, and survivin. For effects on cell invasion, we compared matrix metalloproteinase 2 (MMP-2) and MMP-9. For analyzing **β**-catenin stability, we compared active **β**-catenin (p*-*β**-catenin [S552]), inactive **β**-catenin (p*-*β**-catenin [S33/37, T41]), active AKT (p-AKT [S473]), and inactive GSK3**β** (p-GSK3 [S9]). For synergistic effects, we compared TKI's target RAS/RAF/MEK1/ERK1 axial molecules and active STAT3 (p-STAT3 [Y705]). The immunoreactive bands were revealed by the use of enhanced chemiluminescence (Millipore) then developed and quantified by the use of the UVP BioSpectrum Imaging System (Ultra-Violet Products Ltd.).

### 2.6. Immunohistochemistry and Immunocytochemistry

We used 4 *μ*m sections of xenografted tumors for immunohistochemistry. After blocking with 10% goat serum for 1 hr and incubation with **β**-catenin, Ki-67, and survivin antibodies (1 : 200 each) for 2 hr at room temperature, sections were washed in triplicate with 1xTBST (10 mM Tris pH 7.4, 150 mM NaCl, 0.1% and Tween-20) for 10 min; slides were processed by the use of the UltraVision Quanto Detection System (Thermo Scientific) and counterstained with hematoxylin. For immunocytochemistry, about 2 × 10^4^ cells were seeded on 18 × 18 mm cover glass. After treatment, cells were washed with 1x PBS (200 mM NaCl, 3 mM KCl, 10 mM Na_2_PO_4_, and 1.5 mM KH_2_PO_4_, pH 7.4) twice, fixed in acetone/methanol (1 : 1) at −20°C for 30 min, and permeabilized with 0.1% Triton X-100 in 1x PBS at room temperature for 10 min. Cells were then washed 3 times with 1xTBST and blocked in 10% goat serum for 1 hr. After incubation with the same antibodies (1 : 200) for 2 hr at room temperature, cells were washed 3 times with 1xTBST for 10 min, stained by the use of the UltraVision Quanto Detection System (Thermo Scientific), and counterstained with hematoxylin. Immunohistochemical and immunocytochemical results of each marker were quantified with Aperio ImageScope and Spectrum Software ver. 10.0.

### 2.7. MTT Assay

Cells treated with concentrations of ovatodiolide (0, 0.625, 1.25, 2.5, 5, 10, 20, 40, and 80 *μ*M) and controls were washed twice with 1x PBS and subjected to 3-(4, 5-dimethylthiazol-2-yl)-2,5-diphenyltetrazolium bromide (MTT) assay for detection of cell viability. In brief, 20 *μ*L of 5 mg/mL MTT reagent was added to each well and incubated at 37°C for 3.5 hr before absorbance was read at 570 nm at 0, 24, 48, and 72 hr. Each condition involved 6 repeats. The IC_50_ value for each cell line was determined by the use of CalcuSyn v1.1.1 (Biosoft).

### 2.8. Flow Cytometry

Subconfluent cells were trypsinized, washed with 1x PBS, and adjusted to 2 × 10^6^ cells/mL. A total of 1 × 10^6^ cells were fixed with 100% EtOH for 10 min then incubated with 1 mg/mL propidium iodide for 10 min at room temperature. Cells were analyzed within 20 min by the use of BD FACSCalibur (BD Biosciences).

### 2.9. Cell Migration and Invasion Assay

For wound healing assay, cells were plated in 6-well plates and cultured to 90% confluence. Each RCC cell was treated with 10, 20, or 40 *μ*M ovatodiolide and scraped with a p200 tip (time 0). Before imaging, suspended cells were washed off. The distance of migrating cells was measured from images (5 fields) at 24 and 48 hr after ovatodiolide treatment. The results of wound healing assay were normalized as ratio of wound repaired area to the nontreated control set to 100%.

Transwell assay of each RCC cell was assessed by use of 8 *μ*m inserts (BD Biosciences). In all, 1 × 10^4^ cells were loaded into upper wells, and both upper (200 *μ*L) and lower (1 mL) chambers were filled with complete medium (RPMI-1640 or DMEM supplemented with 10% fetal bovine serum (FBS)) containing 20 *μ*M ovatodiolide or 0.1% DMSO. For invasion assay, each insert was coated with 1 mg/mL Matrigel at 37°C for 5 hr. An amount of 1 × 10^4^ cells was loaded into a coated insert, and both upper (200 *μ*L) and lower (1 mL) chambers were filled with complete medium containing 20 *μ*M ovatodiolide or 0.1% DMSO. The migration and invasion chambers were incubated in a humidified 5% CO_2_ incubator at 37°C for 24 hr. Cells were fixed with 500 *μ*L methanol/acetone (1 : 1) for 15 min; the inner surface of the upper chambers was wiped with a cotton swab to remove the unmigrated cells for migration assay or Matrigel was scraped off for invasion assay. The chambers were washed with 500 *μ*L 1x PBS and stained with 500 *μ*L hematoxylin for 1 min at room temperature. After washing with 1x PBS, the transwell membranes were torn off and placed on slides. The stained cells were analyzed by the use of ImageJ software and 5 random fields were counted at 100x magnification. All data represent the mean of triple independent transwell assays.

### 2.10. Zymography

The enzymatic activities of MMP-2 and MMP-9 were determined by gelatin zymography. In brief, conditioned media were prepared with standard SDS gel-loading buffer containing 0.01% SDS without **β**-mercaptoethanol or DTT and not boiled before loading. An amount of 50 *μ*g conditioned media underwent SDS-PAGE with 0.1% gel. After electrophoresis, gels were washed twice with 1x TBST then incubated with 2% Triton X-100 for 30 min at room temperature to remove SDS, then in 30 mL reaction buffer (40 mM Tris-HCl, pH 8.0, 10 mM CaCl_2_, and 0.02% NaN_3_) for 24 hr at 37°C. Before scanning, gels were stained with Coomassie Brilliant Blue R-250 for 30 min and destained with destaining solution (30% methanol, 10% acetic acid, and 60% water).

### 2.11. Detection of *β*-Catenin Nuclear Translocation and Stability

An amount of 2 × 10^6^ of each RCC cell line was treated with DMSO or ovatodiolide (10, 20, and 40 *μ*M) for 24 hr, and cytoplasmic and nuclear extracts were separated with the use of NE-PER Nuclear and Cytoplasmic Extraction Reagents (Thermo Scientific). In all, 20 *μ*g nuclear and cytoplasmic extracts were used for SDS-PAGE and Western blot analysis to compare *β*-catenin nuclear and cytoplasmic distribution.

*β*-Catenin degradation was well documented with the 26S proteasome pathway. We compared the ovatodiolide effects on *β*-catenin stability in RCC cells by treating cells with 40 *μ*M ovatodiolide and translation inhibitor cyclohexamine (CHX, 100 *μ*g/mL) or 26S proteasome inhibitor MG-132 (10 or 20 *μ*M) for 48 hr, and 20 *μ*g total cell lysates were assessed for SDS-PAGE and western blot analysis.

### 2.12. Endogenous Coimmunoprecipitation

In all, 5 × 10^6^ of Caki-1, 786-O, ACHN, A498, and HEK293T cells were lysed in 0.5% NP-40 protein lysis buffer (50 mM Tris-HCl, pH 7.4, 250 mM NaCl, 0.5% (vol/vol) NP-40, 5 mM EDTA, and 2x proteinase inhibitors) and centrifuged at 16,000 ×g for 10 min at 4°C. The supernatant was incubated with the indicated primary antibody with protein A plus agarose (Invitrogen) for 4hr incubation at 4°C. The beads were washed 6 times with 0.5% NP-40 protein lysis buffer and resuspended in 30 *μ*L SDS loading buffer, boiled, and used for SDS-PAGE and western blot analysis.

### 2.13. Colony-Forming Assay

*In vitro* tumorigenicity was evaluated by colony-forming assay. In brief, 2 mL of 0.5% agarose in complete RPMI-1640 was used as bottom agar in a 6-cm dish, and 2 × 10^4^ cells were mixed with 0.3% agarose in complete RPMI-1640 containing 20 *μ*M ovatodiolide or 0.1% DMSO. Cells were maintained in a humidified 5% CO_2_ incubator at 37°C for 15 days with fresh medium replaced every 3 days. At the 15th day, cells were stained with crystal violet for 1 min and destained with tap water for 15 min. Colonies in each dish were counted by the use of ImageJ with triplicated repeats for each condition.

### 2.14. Xenografting

Female Balb/c nude mice (6 weeks old) were purchased from the National Laboratory Animal Center (Taiwan) and acclimated for 1 week. In brief, 1 × 10^7^ 786-O or ACHN cells in 100 *μ*L of 1x PBS with ~10 mg/mL Matrigel (BD Biosciences) were implanted into the right flank of each mouse. RCC cells at passage 8 were used for xenografting. Before xenografting, cells were tested for mycoplasma by the use of the e-Myco Mycoplasma PCR Detection Kit (Intron). 786-O cells were xenografted into 25 nude mice, and 18 mice showed ~50 mm^3^ tumors after 7 days. ACHN cells were xenografted into 25 nude mice, and 20 mice revealed ~50 mm^3^ tumors after 7 days. We chose 18 mice from each group and randomly divided them into 3 groups (6 mice/group) for systematic treatment: DMSO, 50 mg/kg ovatodiolide, or 100 mg/kg ovatodiolide in 60 *μ*L PBS with 0.5% DMSO by intraperitoneal injection daily. Control mice were intraperitoneally injected with 60 *μ*L PBS and 0.5% DMSO daily. Tumor size was measured every 2 days with the use of calipers and calculated by (length × width^2^)/2. Tumors were removed at 22 days for 786-O cells and 30 days for ACHN cells, because the body weight of some 786-O xenografted mice was lower than the regulation of The Laboratory Animal Center of National Defense Medical Center and mice should be sacrificed. After measuring tumor weight, a small part of each tumor was flash-frozen in liquid nitrogen for western blot analysis and other parts were fixed with formalin for immunohistochemistry.

### 2.15. Statistical Analysis

Real-time PCR data and cell numbers from transwell assay were recorded as continuous data and analyzed by Student's *t*-test. Statistical analyses involved the use of SPSS v16.0 and Microsoft Excel 2007. All statistical tests and *P* values were two sided. *P* < 0.05 was considered statistically significant.

## 3. Results

### 3.1. Screening for *β*-Catenin Signaling Inhibitory Compounds in RCC Cell Lines

**Figure 1:**
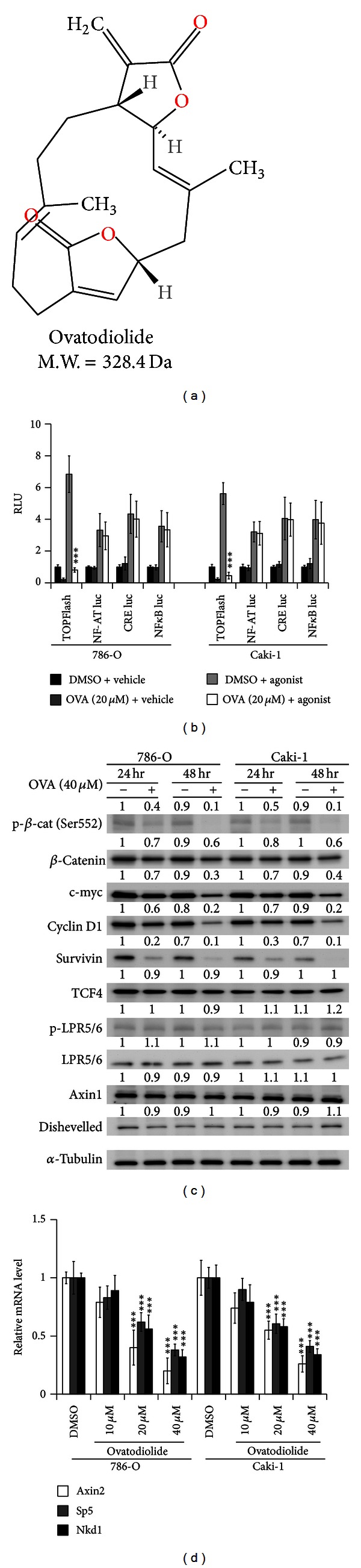
Ovatodiolide inhibits *β*-catenin signaling. (a) The structural formula of ovatodiolide. (b) TOP-flash, NF-AT, CRE, and NF*κ*B reporter assays to evaluate the specificity of ovatodiolide. After 24 hr of transfection with a reporter plasmid, cells were exposed for 24 hr to DMSO or 20 *μ*M ovatodiolide with agonist (LiCl (20 mM) for Top-flash, ionomycin (1 *μ*g/mL) for NF-AT luc, forskolin (10 *μ*M) for CRE luc, and TNF*α* (10 ng/mL) for NF*κ*B luc activity controls) or vehicle control. Luciferase activity was assayed at 48 hr after transfection. (c) Western blot assay of effect of 40 *μ*M ovatodiolide on protein levels of active *β*-catenin (p-*β*-catenin [S552]) and its downstream genes (c-myc, cyclin D1, and survivin) and other WNT molecules (TCF4, LRP5/6, p-LRP5/6, Axin1, and dishevelled) in RCC cell lines 786-O and Caki-1. (d) Quantitative real-time PCR of mRNA levels of *β*-catenin signaling target genes Axin2, Sp5, and Nkd1 in RCC cells after 48 hr of ovatodiolide treatment. All experiments were performed in triplicate. Data are mean ± SD from triplicate experiments. *P < 0.05, **P < 0.01, and ***P < 0.001.

To identify potential compounds suppressing **β**-catenin signaling activity, we performed a two-step screening of 21 pure compounds of *C. reticulata* Blanco, 16 compounds of *H. syriacus* L., and 23 compounds of *A. indica* L. The first step consists of *in silico* drug screening involving the PubChem Compound database to search for human tumor cell line growth inhibition/antiproliferative activity, *in vivo* antitumor/anticancer activity, induction of apoptosis, or cytotoxicity (summarized in Table S1). In all, 11 compounds were selected, including 5 pure compounds of *C. reticulata* Blanco, 4 compounds of *H. syriacus* L., and 2 compounds of *A. indica* L. In the second step, these 11 compounds were used to examine *β*-catenin signaling activity by the use of the TCF/LEF reporter assay with 786-O and Caki-1 RCC cell lines, which feature high endogenous *β*-catenin signaling. Psoralen, a pure compound of *Psoralea corylifolia* L., was used as a *β*-catenin signaling inductive control [34]. Ovatodiolide (Figure 1, CAS no.: 3484-37-5, structure obtained from ChemSpider) had the highest inhibitory efficiency for luciferase activity, and the TOP/FOP luciferase ratios were reduced 89% and 92% after 24 hr treatment of 786-O and Caki-1 cells, respectively, with 40 *μ*M ovatodiolide (Figure S1A). Ovatodiolide, 40 *µ*M, cotreated with rhWNT3a or LiCl significantly decreased rhWNT3a or LiCl-induced *β*-catenin signaling, respectively, with TOP/FOP ratios reduced ~80% in both cell lines, respectively (Figure S1B).

To confirm the specificity of ovatodiolide in suppressing *β*-catenin signaling, we compared the ovatodiolide effects with NF-AT, CRE, and NF*κ*B luciferase reporter assays, with their agonistic compounds used as inductive controls. Ovatodiolide specifically inhibited the luciferase activity of TOP-flash but had no effect in NF-AT, CRE, and NF*κ*B reporters (Figure 1(b)). The suppressive effects of ovatodiolide were further evaluated with *β*-catenin/TCF/LEF downstream genes by immunocytochemistry. The staining for nuclear *β*-catenin and its downstream genes cyclin D1 and survivin was less in ovatodiolide-treated RCC cells than in DMSO vehicle controls (Figure S1C). In 4 RCC cell lines (786-O, Caki-1, A498, and ACHN), ovatodiolide reduced levels of active *β*-catenin (Ser552 phosphorylation on *β*-catenin) and its downstream genes (c-myc, cyclin D1, and survivin) but not other WNT molecules (TCF4, LRP5/6 and its active phosphorylated form, Axin1, and disheveled) (Figures 1(c) and S1D). However, ovatodiolide had no inhibitory effects in HEK293T, a low constitutive WNT signaling cell, or in normal kidney epithelial HK-2 cells (Figure S1D). Ovatodiolide treatment at 10, 20, and 40 *μ*M reduced mRNA levels of *β*-catenin-signaling target genes Axin2, Sp5, and Nkd1 [35] by 60% to 80% in both RCC cells (Figure 1(d)).

### 3.2. Ovatodiolide Reduces Cell Viability and Induces Apoptosis in RCC Cells

**Figure 2:**
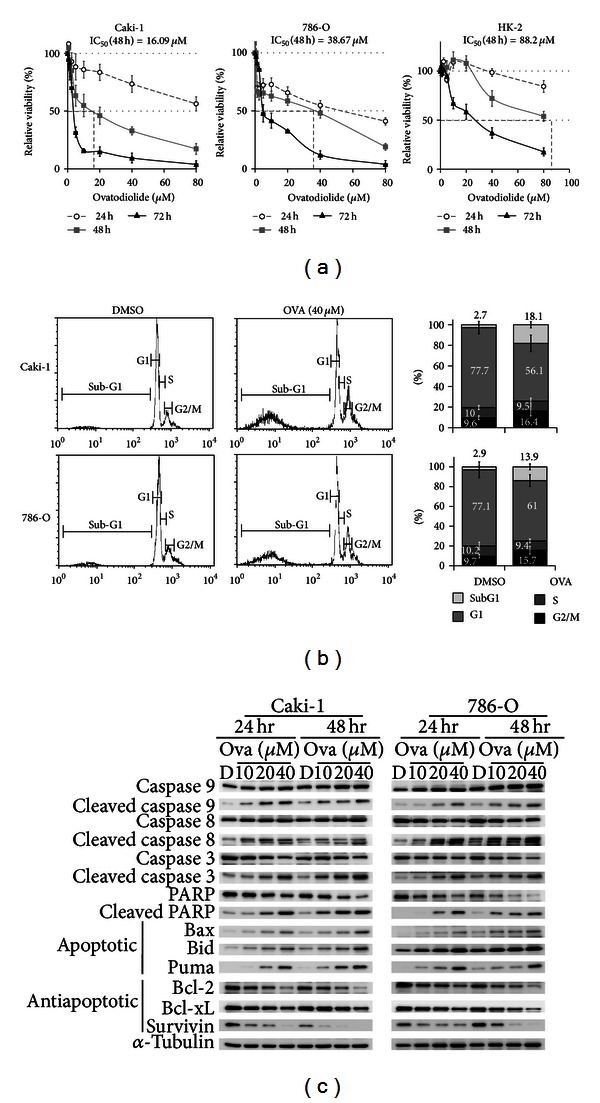
Ovatodiolide inhibits RCC cell viability and induces apoptosis. (a) The effect of ovatodiolide on cell viability was evaluated in 786-O, Caki-1, and HK-2 cells by MTT assay at various times. The IC_50_ at 48 hr was calculated, and the relative viability was the percentage of MTT absorbance of ovatodiolide to DMSO-treated cells. Data are mean ± SD of triplicate experiments. (b) Flow cytometry of the effect of ovatodiolide on cell cycle distribution. 786-O and Caki-1 cell lines were treated with 40 *μ*M ovatodiolide for 48 hr, and sub-G1 and G2/M populations were measured. Data are mean ± SD of triplicate experiments. (c) Western blot analysis of protein levels of cleaved caspase 8 and 9, and apoptotic proteins Bax, Bid and, PUMA, and antiapoptotic proteins Bcl-2, Bcl-xL, and survivin in 786-O and Caki-1 cell lines treated with ovatodiolide for 24 or 48 hr.

To evaluate the cytotoxicity of ovatodiolide in RCC and normal kidney cell lines, we analyzed cell viability. Ovatodiolide had a significantly higher cytotoxic effect in 4 RCC cell lines (786-O, Caki-1, ACHN, and A498) but less effect in HK-2 cells (Figures 2(a), S2A, and S2B). The IC_50_ with 48 hr treatment for HK-2 cells was 88.20 *μ*M, which is much higher than that for RCC cells (16.09, 38.6, 28.40, and 25.81 *μ*M for Caki-1, 786-O, ACHN, and A498 cells). With 48 hr treatment, ovatodiolide significantly increased the sub-G1 cell population by ~5- to 6-fold in RCC cells than in controls (Figures 2(b) and S2C). G2/M arrest was increased ~1.5-fold in ovatodiolide-treated cells, perhaps associated with survivin downregulation [36, 37]. The apoptosis-inductive effects were also confirmed; cleaved caspase 3 and cleaved PARP level were markedly increased in ovatodiolide-treated cells because of the induction of both intrinsic and extrinsic apoptotic pathways (Figures 2(c) and S2D); cleaved caspase 9 and 8 levels were increased and therefore upregulated apoptotic proteins (Bax, Bid, and PUMA) and downregulated antiapoptotic proteins (Bcl-2, Bcl-xL, and survivin) (Figures 2(c) and S2D). To avoid the ovatodiolide inhibitory effect on *β*-catenin signaling (Figure 1(c)) was a result of high-dose (exceeded the IC_50_) induced cell apoptosis, a sub- IC_50_ concentration (15 *μ*M) was also examined in Caki-1 and 786-O for 24 h and 48 h. As in Figure S2E, 15 *μ*M ovatodiolide also reduced levels of active *β*-catenin (p-Ser552-*β*-catenin) and its downstream genes (c-myc, cyclin D1, and survivin) but not other WNT molecules (TCF4), LRP5/6 and its active phosphorylated form, Axin1, and dishevelled.

### 3.3. Ovatodiolide Reduced RCC Aggressiveness by Suppressing *β*-Catenin Signaling

**Figure 3:**
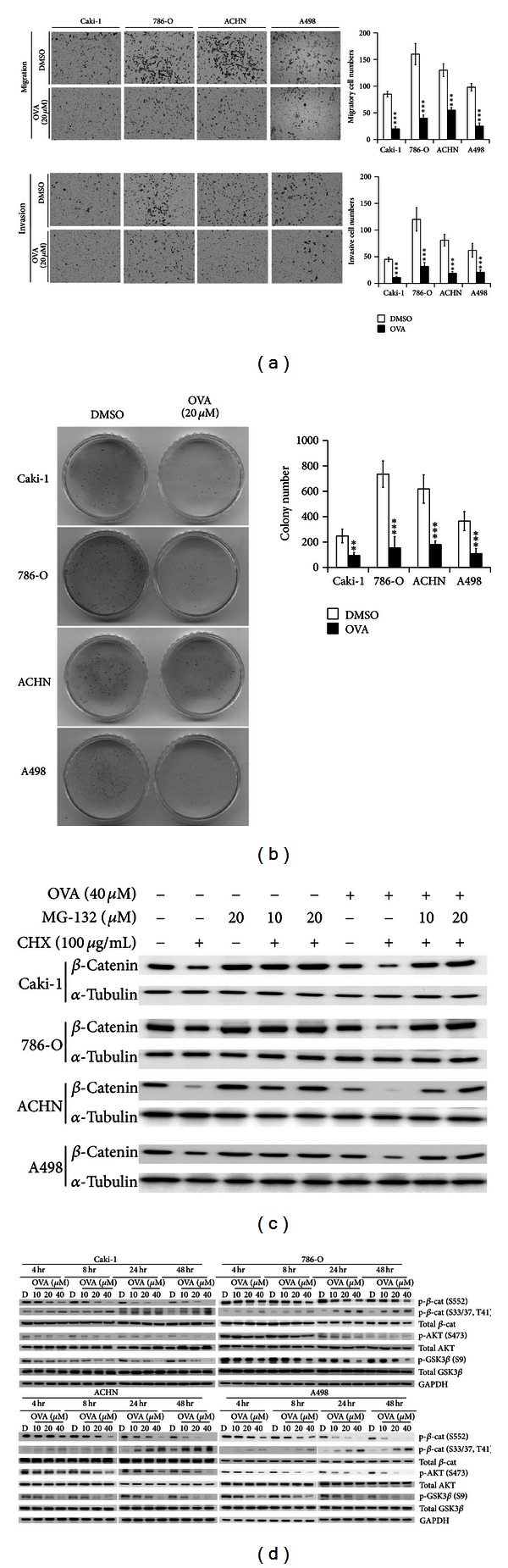
Ovatodiolide inhibits RCC cell migration, invasion, and tumorigenicity. (a) Transwell assay of cell migratory ability (upper) and invasive ability (lower) in four RCC cell lines. Five random fields of each transwell membrane were counted at 100x magnification. Data are mean ± SD of triplicate independent repeats. (b) Colony-forming assay of* in vitro* tumorigenicity. Data are mean ± SD of triplicate experiments. (c) Effect of ovatodiolide (40 *μ*M) and cyclohexamine (CHX, 100 *μ*g/mL) or MG-132 (10 or 20 *μ*M) for 48 hr. (d) RCC cells were treated with doses of ovatodiolide or DMSO for 48 hr. Western blot analysis of phosphorylation of AKT (p-AKT [S473]) and inhibitory regulation of p-GSK-3*β* (S9) and *β*-catenin (p-*β*-catenin [S552]) and inactive form of *β*-catenin (phosphorylation at S33/37, T41) in four RCC cells.

To examine the inhibitory effects of ovatodiolide on RCC aggressiveness, we evaluated its effects on cell migration, invasion, and tumorigenicity. After 48 hr of 40 *μ*M ovatodiolide treatment, migratory ability was reduced >50% in each RCC cell line as compared with controls (Figure S3A). Similar inhibitory effects were observed by transwell assay (Figure 3(a) upper). For cell invasion, 24 hr ovatodiolide treatment significantly reduced >65% of invasive cell numbers as compared with controls (Figure 3(a) lower). Ovatodiolide treatment reduced the protein expression of invasion factors MMP-2 and MMP-9 and therefore reduced their digestive activities (Figure S3B).

**Figure 4:**
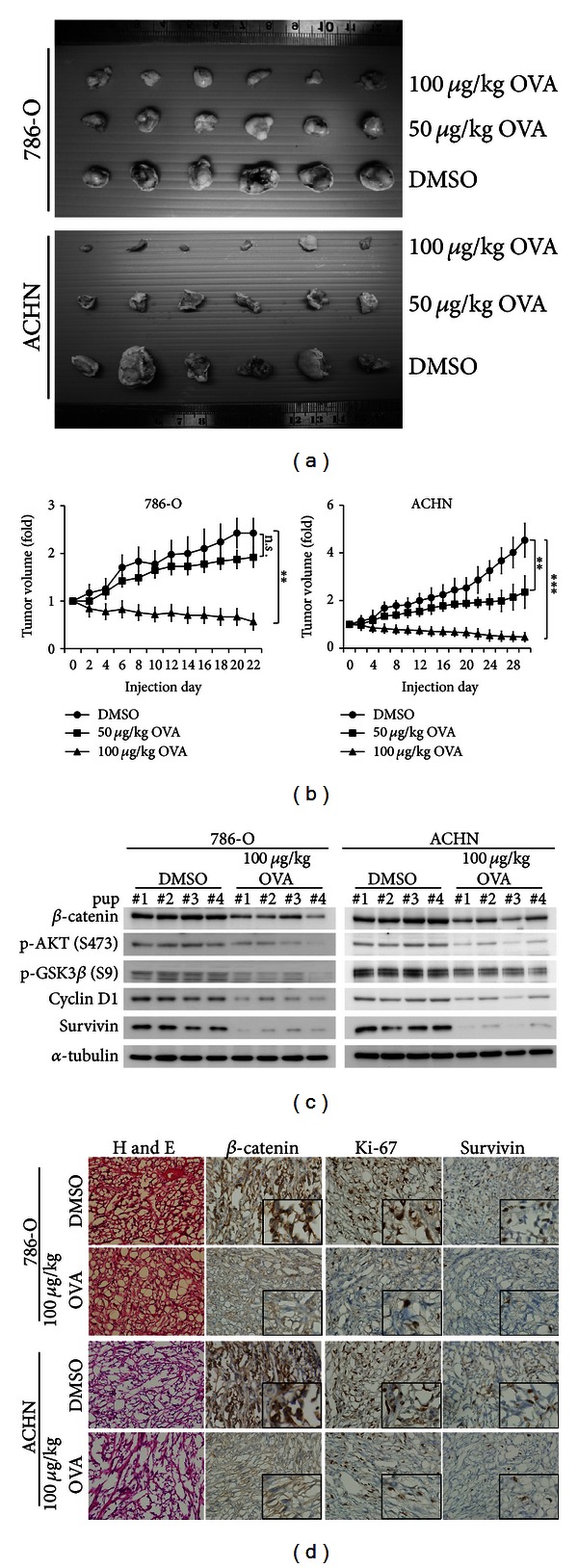
Ovatodiolide inhibits *in vivo* tumorigenicity. (a) 786-O and ACHN cells were xenografted each in six mice. Xenografted mice were treated with 50 *μ*g/kg, 100 *μ*g/kg ovatodiolide or DMSO control. (b) Tumor volume of 50 *μ*g/kg, 100 *μ*g/kg ovatodiolide treatment or DMSO control. Data are mean ± SD of triplicate experiments. (c) Western blot analysis of protein levels of *β*-catenin, p-AKT (S473), p-GSK3*β* (S9), cyclin D1 and survivin with ovatodiolide (100 *μ*g/kg). (d) Immunohistochemistry of *β*-catenin nuclear translocation and quantity in mice, and cell cycle (Ki-67 level) and survival (survivin level).

Tumorigenicity of ovatodiolide was evaluated with *in vitro* colony-formation assay and *in vivo* xenografting. Treatment with 20 *μ*M ovatodiolide for 20 days significantly reduced colony forming ability ~60 to 80% in cell lines (Figure 3(b)). Balb/c nude mice were subcutaneously injected with 1 × 10^7^ 786-O or ACHN cells, two higher tumorigenic RCC cell lines. Tumor size reached ~50 mm^3^ after 7 days. Intraperitoneal injection of 50 or 100 *μ*g/kg for 22 days in mice with 786-O xenografts and 30 days in mice with ACHN xenografts, hence, systematic treatment, was a prior way for the smallest molecule drug delivery. Ovatodiolide significantly reduced *in vivo* tumorigenicity of 786-O or ACHN cells, especially with 100 *μ*g/kg ovatodiolide (Figures 4(a) and S4A). Treatment with 100 *μ*g/kg ovatodiolide significantly reduced both tumor volume and tumor weight compared to controls (Figures 4(b) and S4B). Ovatodiolide-treated mice showed no distinguishable body weight loss or systemic toxicity (Figures S4B and S4D). However, in 786-O-xenografted mice, DMSO significantly reduced body weight after 17 days of 786-O cell injection (Figure S4C).

### 3.4. Ovatodiolide Reduced *β*-Catenin Stability by Inhibiting AKT Activation and Reducing GSK3*β* Phosphorylation

To explore the ovatodiolide inhibition of *β*-catenin signaling, we further investigated its effects on *β*-catenin stability and related regulatory molecules. Ovatodiolide treatment did not modify the mRNA level *β*-catenin in each RCC cell (Figure S3C). However, *β*-catenin nuclear translocation was dose-dependently decreased after 24 hr ovatodiolide treatment (Figure S3D). Consequently, RCC cells were cotreated with ovatodiolide, the translation inhibitor CHX, and 26S proteosome inhibitor MG-132 to confirm the suppression of *β*-catenin stability. Cotreatment with CHX decreased most of the *β*-catenin protein level, and MG-132 treatment abrogated this inhibitory effect of ovatodiolide (Figure 3(c)). Ovatodiolide promoted *β*-catenin degradation through the 26S proteosome pathway but not lysosome-associated protein degradation pathway (Figure S3E).

The interaction between E-cadherin, *β*-catenin, TCF4, and *β*-catenin was further compared by coimmunoprecipitation. TCF4-*β*-catenin interaction but not E-cadherin-*β*-catenin interaction was remarkably reduced in each cell (Figure S3F). Despite the Caki-1, 786-O, and A498 being so-called E-cadherin-negative cells, the faintly immunoprecipitated E-cadherins were unchanged after ovatodiolide treatment. *β*-Catenins phosphorylated by GSK3*β* at residues T41, S37, and S33 are recognized by the *β*-TrCP E3 ubiquitin-ligase complex, ubiquitinylated, and ultimately degraded by the 26S proteosome [38]. GSK3*β* (S9) phosphorylated by active AKT (i.e., AKT S473 phosphorylated form) inhibits GSK3*β* kinase activity [39]. Otherwise, *β*-catenin phosphorylated at S552 by active AKT enhances *β*-catenin protein levels and nuclear signaling [40, 41]. We addressed these possible regulators and **β**-catenin phosphorylation status with ovatodiolide treatment. Ovatodiolide dose- and time-dependently reduced both phosphorylated AKT (S473) and GSK3*β* (S9) levels (Figure 3(d)). Therefore, phosphorylated *β*-catenin S552 forms were decreased but phosphorylated S33/37/T41 forms were increased. Treatment with the AKT inhibitor VIII (5 *μ*M) induced similar effects, and constitutively active AKT abrogated the ovatodiolide-induced inhibition of *β*-catenin signaling (Figures S5A and S5B). The effect of constitutively active Akt also partially rescued the OVA-induced cell death (Figure S6A). Besides, ovatodiolide treatment did not modify other downstream molecules of AKT, including p-Foxo3a (T32), p-mTOR (S2448), and p-p70S6K (T389) levels (Figure S5A). Therefore, ovatodiolide inhibited *β*-catenin signaling by reducing *β*-catenin activity and stability. With ovatodiolide treatment of xenografted mice, levels of phosphorylated *β*-catenin, cell cycle markers Ki-67 and cyclin D1, and survival marker survivin were decreased as compared with controls (Figures 4(c) and 4(d)) and levels of phosphorylated AKT (S473) and GSK3*β* (S9) were decreased (Figure 4(c)). Thus, ovatodiolide reduced *β*-catenin signaling* in vivo *and reduced RCC cell tumorigenicity. The physical binding between ovatodiolide and *β*-catenin was simulated on the molecular docking website PATCHDOCK with the 3D structure files for ovatodiolide (PubChem: CID_6451060) and *β*-catenin (PDB: 1QZ7). As in Figure S6B, the ovatodiolide inserted into the *β*-catenin molecule enclosing by the AKT phosphorylation site, Ser-552 residue, and may result in a stereochemical change to reduce its activation. However, there is no proper 3D structure including N-terminus of *β*-catenin and it is uneasy to evaluate whether ovatodiolide also bound to the GSK3*β* targeting Ser33, Ser37, or Thr41 residues.

### 3.5. Ovatodiolide Synergistically Increased Sensitivity of RCC Cells *In Vitro* with Sorafenib or Sunitinib Treatment

**Figure 5:**
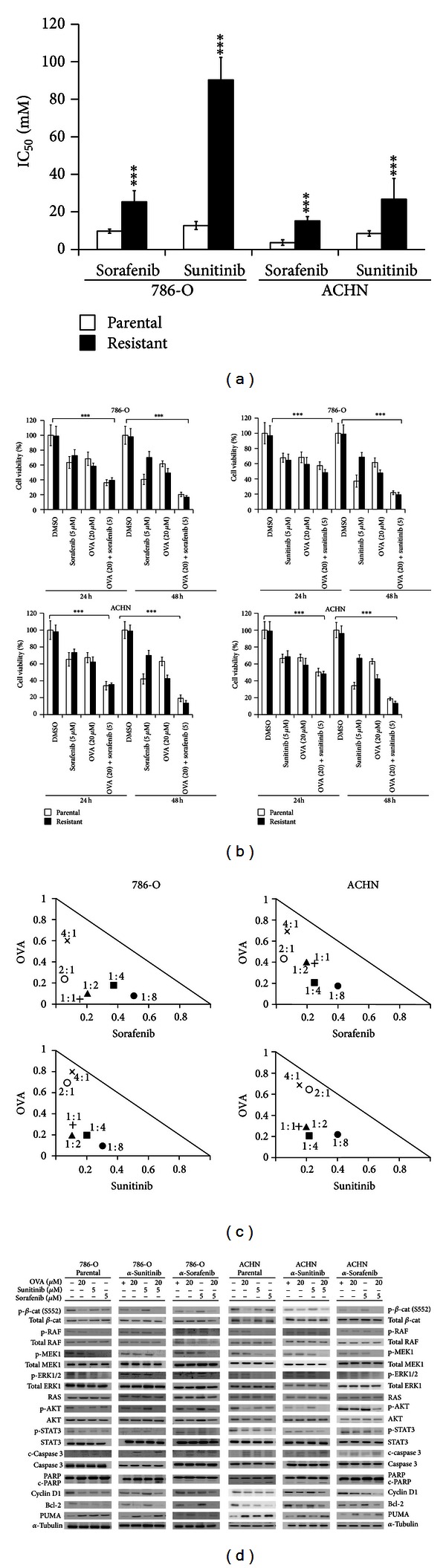
Ovatodiolide in combination therapy and against TKI-resistant RCC cell lines. (a) The half maximal inhibitory concentration of the conventional chemotherapy drugs sorafenib and sunitinib in RCC cells. Data are mean ± SD. *P < 0.05, **P < 0.01, and ***P < 0.001. (b) Cell viability assay of both parental and TKI-resistant 786-O and ACHN cells treated with 20 *μ*M ovatodiolide, 5 *μ*M sorafenib or sunitinib, or their combination for 24 and 48 hr. Data are mean ± SD. *P < 0.05, **P < 0.01, and ***P < 0.001. (c) Isobologram analysis of the combination of ovatodiolide and sorafenib or sunitinib. Symbols designate the combination index value for each fraction affected. The curves were generated by the use of CalcuSyn software to fit the experimental points. Data are representative of 3 independent experiments. Values below the line are synergistic, those close to the line are additive, and those above the line are antagonistic. (d) Western blot analysis of the combined effect of ovatodiolide (20 *μ*M) with sorafenib (5 *μ*M) or sunitinib (5 *μ*M) for 48 hr comparing the parental, anti-sunitinib (*α*-sunitinib), and anti-sorafenib (*α*-sorafenib) 786-O or ACHN cells. Evaluation included TKIs targeting RAS-RAF-MEK1-ERK1 signaling and pSTAT3 status and ovatodiolide-targeted p-*β*-catenin (S552). Cytotoxicity was compared by levels of apoptotic cleaved caspase 3 and PARP, antiapoptotic Bcl-2, and apoptotic PUMA and cell cycle cyclin D1.

We cultured sorafenib-resistant or sunitinib-resistant 786-O and ACHN cell lines to determine whether ovatodiolide could resensitize drug-resistant cells towards these chemotherapeutic agents. On treatment with 5 *μ*M sorafenib or sunitinib for 48 hr, all drug-resistant 786-O and ACHN cells showed at least 2.6-fold significantly greater IC_50_ than their parental cells (Figure 5(a)). Drug-resistant cells showed greater viability (Figure 5(b)) and increased levels of cyclin D1 and antiapoptotic Bcl-2 but also lower levels of apoptotic proteins (cleaved caspase 3, cleaved PARP, and PUMA) as compared to their parental cells. Combined ovatodiolide and sorafenib or sunitinib treatment significantly increased the cytotoxic effect in both drug-resistant 786-O and ACHN cells as compared with their treatment alone (Figure 5(b)).

Assessment of the synergistic activity of 20 *μ*M ovatodiolide with 5 *μ*M sorafenib or sunitinib involved the isobolographic method [42] for drug-resistant 786-O and ACHN cells (Figure 5(c)). To confirm that the synergistic cytotoxicity was caused by specific inhibition of receptor tyrosine kinase and *β*-catenin signaling, we compared the inhibitory effects of these drugs combined on the RAS-RAF-MEK1-ERK1 signaling pathway, a typical target of sorafenib or sunitinib, and the AKT-GSK3**β**-**β**-catenin axis. Ovatodiolide with sorafenib or sunitinib synergistically reduced levels of phosphorylated RAF1, MEK1, and ERK1 in drug-resistant 786-O and ACHN cells (Figure 5(d)). Combined treatment synergistically reduced phosphorylation of *β*-catenin (S552). STAT3 (Y775) is another target of sorafenib or sunitinib [43]. Phosphorylation of STAT3 (Y775) was also reduced with ovatodiolide combined with sorafenib or sunitinib. In addition, ovatodiolide alone inhibited *β*-catenin signaling without affecting RAS-RAF-MEK-ERK signaling or STAT3 status. Ovatodiolide conferred a synergistic effect that resensitized sorafenib- or sunitinib-resistant cells towards these chemotherapeutic agents.

## 4. Discussion

This study demonstrated that ovatodiolide is an anti-*β*-catenin signaling compound, at least in RCC, as evidenced by its ability to reduce *β*-catenin stability and suppress *β*-catenin activation *in vitro* and *in vivo*. More importantly, when combined with sorafenib or sunitinib, ovatodiolide could enhance the treatment response and overcome drug resistance. We successfully used compound screening with the PubChem BioActivity database combined with TOP/FOP reporter assays to target *β*-catenin signaling in RCC with ovatodiolide. And this procedure should be easily and quickly possessed for screening of specific signaling inhibitors among most purified compounds or synthetic chemicals.

Ovatodiolide is a cemsbrane-type diterpenoid and one of the major components of *A. indica* L. It can reduce lipopolysaccharide-induced nitric oxide and cytokine levels in macrophages [44] and blood pressure in anaesthetized dogs [45] and is responsible for the anti-inflammatory and antihypotensive effects of *A. indica*. Ovatodiolide also has cytotoxic effects in some human cancer cell lines by inducing apoptotic pathways [46, 47] and has antimetastatic effects by downregulating c-Jun N-terminal kinase, p38 mitogen-activated protein kinase, and PI3K/AKT signaling pathways, therefore inhibiting NF*κ*B-MMP-9 axis activation [48].

Several drugs and synthetic or natural compounds have been reported to inhibit and/or modulate *β*-catenin signaling [49, 50]; however, their detailed mechanisms are little understood. These small-molecule inhibitors may act by reducing *β*-catenin stability [51], blocking *β*-catenin-TCF interaction [52, 53] or *β*-catenin-CREB binding protein interaction [54], stabilizing Axin2 level [55], preventing dishevelled-Frizzled interaction [56], or other indirect inhibition [50]. Here, we found that ovatodiolide reduced phosphorylation of AKT (S473) and therefore downregulated the phosphorylation of its downstream molecules GSK3*β* (S9) and *β*-catenin (S552) in RCC cell lines. Reduced phosphorylation of GSK3*β* (S9) prolongs GSK3*β* activation [39] and decreases *β*-catenin protein stability. Reduced phosphorylation of *β*-catenin (S552) inhibits *β*-catenin activation and nuclear signaling [40, 41]. We found that ovatodiolide significantly downregulated survivin in RCC cells both *in vitro* and *in vivo*. As in Figure S5C, schematic diagram depicts the mechanism of inactivation of *β*-catenin by ovatodiolide in RCC cells. Survivin knockdown has been associated with G2/M arrest [36, 37, 57], which may explain why *β*-catenin signaling inhibitors induced G2/M arrest [58], although it has also been associated with Axin2 reduction [42].

Combined treatment with ovatodiolide and sorafenib or sunitinib overcame drug resistance in TKI-resistant RCC cells. Sorafenib and sunitinib are approved for treatment of advanced RCC by the US Food and Drug Administration. Both have been reported as multikinase inhibitors of vascular endothelial growth factor receptor, platelet-derived growth factor receptor, RAF, and several different tyrosine kinases and therefore are used in treating several cancers [59]. RCC patients treated with these TKIs show prolonged progression-free survival and/or overall survival, but resistance to therapy is inevitable [60]. Despite permanent genetic or epigenetic changes in the tumor or selection of drug-resistant clones, resistance of these TKIs is attributed to resumption of angiogenesis, or “angiogenic escape,” according to reversible gene expression in the tumor and/or its microenvironment [61] or accompanied by alternative signaling pathways such as reduction in level of interferon **γ**-related angiostatic chemokines [62]. Current strategies to maximize the effectiveness of treatment have mostly focused on sequential, combination, adjuvant, or novel targeted therapy [63].

We also found that RAS-RAF-MEK1-ERK1 signaling is reversibly expressed in sorafenib- or sunitinib-resistant 786-O and ACHN cells. In addition, we found reversed phosphorylated STAT3 status, another target of these TKIs in different cancer types [43]. After combined treatment of ovatodiolide with sorafenib or sunitinib, the reversible gene expression was abrogated and the cytotoxic response was greater than in controls. The *β*-catenin signaling dysregulation involved in the drug resistance of RCC cells [64] and the synergistic effects of *β*-catenin signaling inhibitors and TKIs may be useful to enhance the treatment response of highly chemorefractory, advanced RCC. For evaluation of bioavailability in human, the human equivalent dose (HED) [65] was calculated as follows: HED (mg/kg) = 0.1 mg/kg × 3 (mouse Km)/37 (adult human Km) = 0.008 mg/kg = 0.486 mg/60 kg adult human. And it may be achievable for ovatodiolide treatment in an adult human with a quaque die administration.

In conclusion, ovatodiolide is a potent inhibitor of *β*-catenin signaling and therefore inhibits cell viability, migration, invasion, and both *in vitro* and* in vivo* tumorigenicity of RCC but induces less cytotoxicity in normal kidney cells. Ovatodiolide had synergistic effects with sorafenib or sunitinib and enhanced the combined treatment response. Ovatodiolide may be a promising candidate for RCC treatment.

## Supplementary Material

Ovatodiolide specifically inhibits WNT/*β*catenin signaling and therefore reduces TOP/FOP ratio (see Figure S1A-B), *β*catenin activation and downstream gene expression (see Figure S1C-D). Its purity was also examined (see Figure S1E). Ovatodiolide inhibits RCC cell viability and induces apoptosis were confirmed in all four RCC cells and normal HK-2 cell lines (see Figure S1A-D). And sub-IC50 dose also resulted in similarly inhibitory effects (see Figure S1E). Ovatodiolide inhibitory abilities on cell migration, invasion and tumorigenicity were also confirmed in all four RCC cells (see Figure S3A-B). Ovatodiolide reduces *β*catenin stability and activation on a 26S proteasome dependent manner (see Figure S3C-F). Ovatodiolide significantly suppresses in vivo tumorigenicity without recognizably systemic toxicity (see Figure S4A-D). AKT inhibitor treatment induced effects similar to that of ovatodiolide and constitutively active AKT abrogated the ovatodiolide-induced inhibition of WNT/*β*-catenin signaling (see Figure S5A-C and S6A). According to molecular docking simulation, ovatodiolide inserted into *β*-catenin enclosing Ser-552 residue, the AKT phosphorylation site, and may result in a stereochemical change to reduce its activation.

## References

[1] Ljungberg B, Campbell SC, Choi HY (2011). The epidemiology of renal cell carcinoma. *European Urology*.

[2] Bureau of Health Promotion http://www.bhp.doh.gov.tw/BHPnet/English/Class.aspx?Sub=statistics&No=200705180025.

[3] Weikert S, Ljungberg B (2010). Contemporary epidemiology of renal cell carcinoma: perspectives of primary prevention. *World Journal of Urology*.

[4] Finley DS, Pantuck AJ, Belldegrun AS (2011). Tumor biology and prognostic factors in renal cell carcinoma. *The Oncologist*.

[5] Singer EA, Gupta GN, Srinivasan R (2012). Targeted therapeutic strategies for the management of renal cell carcinoma. *Current Opinion in Oncology*.

[6] Eisen T, Sternberg CN, Robert C (2012). Targeted therapies for renal cell carcinoma: review of adverse event management strategies. *Journal of the National Cancer Institute*.

[7] EU SmPC http://www.ema.europa.eu/humandocs/Humans/EPAR/nexavar/nexavar.htm.

[8] EU SmPC http://www.ema.europa.eu/humandocs/Humans/EPAR/votrient/votrient.htm.

[9] EU SmPC http://www.ema.europa.eu/humandocs/Humans/EPAR/sutent/sutent.htm.

[10] EU SmPC http://www.ema.europa.eu/humandocs/Humans/EPAR/torisel/torisel.htm.

[11] EU SmPC http://www.ema.europa.eu/humandocs/Humans/EPAR/avastin/avastin.htm.

[12] EU SmPC http://www.ema.europa.eu/humandocs/Humans/EPAR/afinitor/afinitor.htm.

[13] Nusse R (2005). Wnt signaling in disease and in development. *Cell Research*.

[14] Saini S, Majid S, Dahiya R (2011). The complex roles of Wnt antagonists in RCC. *Nature Reviews Urology*.

[15] Hsu RJ, Ho JY, Cha TL (2012). WNT10A plays an oncogenic role in renal cell carcinoma by activating *β*-catenin pathway. *PLoS ONE*.

[16] Bilim V, Kawasaki T, Katagiri A, Wakatsuki SJ, Takahashi K, Tomita Y (2000). Altered expression of *β*-catenin in renal cell cancer and transitional cell cancer with the absence of *β*-catenin gene mutations. *Clinical Cancer Research*.

[17] Klaus A, Birchmeier W (2008). Wnt signalling and its impact on development and cancer. *Nature Reviews Cancer*.

[18] Kim YS, Kang YK, Kim JB, Han SA, Kim K, Paik SR (2000). *β*-catenin expression and mutational analysis in renal cell carcinomas. *Pathology International*.

[19] Bruder E, Moch H, Ehrlich D (2007). Wnt signaling pathway analysis in renal cell carcinoma in young patients. *Modern Pathology*.

[20] Guillén-Ahlers H (2008). Wnt signaling in renal cancer. *Current Drug Targets*.

[21] Hirata H, Hinoda Y, Nakajima K (2009). Wnt antagonist gene polymorphisms and renal cancer. *Cancer*.

[22] Barker N, Clevers H (2006). Mining the Wnt pathway for cancer therapeutics. *Nature Reviews Drug Discovery*.

[23] Anastas JN, Moon RT (2013). WNT signalling pathways as therapeutic targets in cancer. *Nature Reviews Cancer*.

[24] Majid S, Saini S, Dahiya R (2012). Wnt signaling pathways in urological cancers: past decades and still growing. *Molecular Cancer*.

[25] Chun G, Lee YB, Ann CH Abstract #3562: characterization of a novel small molecule with potent anticancer activity. http://www.rexahn.com/pdf/newwww/AACR%20poster%20RX-8243%20-Final.pdf.

[26] Fiskus W, Smith J, Mudunuru U (2011). Abstract C144: treatment with *β*-catenin antagonist BC2059 exhibits single agent efficacy and exerts superior activity with tyrosine kinase inhibitor (TKI) or histone deacetylase (HDAC) inhibitor against human AML, CML, and myeloproliferative neoplasm (MPN) progenitor cells. *Molecular Cancer Therapeutics*.

[27] Motzer RJ, Hutson TE, Tomczak P (2009). Overall survival and updated results for sunitinib compared with interferon alfa in patients with metastatic renal cell carcinoma. *Journal of Clinical Oncology*.

[28] Sternberg CN, Davis ID, Mardiak J (2010). Pazopanib in locally advanced or metastatic renal cell carcinoma: results of a randomized phase III trial. *Journal of Clinical Oncology*.

[29] Hudes G, Carducci M, Tomczak P (2007). Temsirolimus, interferon alfa, or both for advanced renal-cell carcinoma. *The New England Journal of Medicine*.

[30] Escudier B, Pluzanska A, Koralewski P (2007). Bevacizumab plus interferon alfa-2a for treatment of metastatic renal cell carcinoma: a randomised, double-blind phase III trial. *The Lancet*.

[31] Rini BI, Flaherty K (2008). Clinical effect and future considerations for molecularly-targeted therapy in renal cell carcinoma. *Urologic Oncology: Seminars and Original Investigations*.

[32] Heng DY, Mackenzie MJ, Vaishampayan U Primary anti-VEGF-refractory metastatic renal cell carcinoma (mRCC): clinical characteristics, risk factors, and subsequent therapy.

[33] Rao YK, Lien HM, Lin YH (2012). Antibacterial activities of *Anisomeles indica* constituents and their inhibition effect on *Helicobacter pylori*-induced inflammation in human gastric epithelial cells. *Food Chemistry*.

[34] Tang DZ, Yang F, Yang Z (2011). Psoralen stimulates osteoblast differentiation through activation of BMP signaling. *Biochemical and Biophysical Research Communications*.

[35] Waaler J, Machon O, von Kries JP (2011). Novel synthetic antagonists of canonical Wnt signaling inhibit colorectal cancer cell growth. *Cancer Research*.

[36] Rödel F, Hoffmann J, Distel L (2005). Survivin as a radioresistance factor, and prognostic and therapeutic target for radiotherapy in rectal cancer. *Cancer Research*.

[37] Sarthy AV, Morgan-Lappe SE, Zakula D (2007). Survivin depletion preferentially reduces the survival of activated K-Ras-transformed cells. *Molecular Cancer Therapeutics*.

[38] Verheyen EM, Gottardi CJ (2010). Regulation of Wnt/*β*-catenin signaling by protein kinases. *Developmental Dynamics*.

[39] Cross DAE, Alessi DR, Cohen P, Andjelkovich M, Hemmings BA (1995). Inhibition of glycogen synthase kinase-3 by insulin mediated by protein kinase B. *Nature*.

[40] Tian Q, Feetham MC, Tao WA (2004). Proteomic analysis identifies that 14-3-3*ζ* interacts with *β*-catenin and facilitates its activation by Akt. *Proceedings of the National Academy of Sciences of the United States of America*.

[41] Fang D, Hawke D, Zheng Y (2007). Phosphorylation of *β*-catenin by AKT promotes *β*-catenin transcriptional activity. *The Journal of Biological Chemistry*.

[42] Chou TC, Talalay P (1984). Quantitative analysis of dose-effect relationships: the combined effects of multiple drugs or enzyme inhibitors. *Advances in Enzyme Regulation*.

[43] Yang F, van Meter TE, Buettner R (2008). Sorafenib inhibits signal transducer and activator of transcription 3 signaling associated with growth arrest and apoptosis of medulloblastomas. *Molecular Cancer Therapeutics*.

[44] Rao YK, Fang SH, Hsieh SC, Yeh TH, Tzeng YM (2009). The constituents of *Anisomeles indica* and their anti-inflammatory activities. *Journal of Ethnopharmacology*.

[45] Momose Y, Nimura M, Arisawa M, Hayashi T, Takeda R, Nakanishi S (1994). Hypotensive activity of ovatodiolides isolated from a Chinese crude drug ‘Fang Feng Cao’. *Phytotherapy Research*.

[46] Arisawa M, Nimura M, Fujita A, Hayashi T, Morita N, Koshimura S (1986). Biological active macrocyclic diterpenoids from Chinese drug “Fang Feng Cao”; II. Derivatives of ovatodiolids and their cytotoxity. *Planta Medica*.

[47] Chen YL, Lan YH, Hsieh PW (2008). Bioactive cembrane diterpenoids of anisomeles indica. *Journal of Natural Products*.

[48] Hou YY, Wu ML, Hwang YC, Chang FR, Wu YC, Wu CC (2009). The natural diterpenoid ovatodiolide induces cell cycle arrest and apoptosis in human oral squamous cell carcinoma Ca9-22 cells. *Life Sciences*.

[49] Moon RT, Kohn AD, de Ferrari GV, Kaykas A (2004). WNT and *β*-catenin signalling: diseases and therapies. *Nature Reviews Genetics*.

[50] Takahashi-Yanaga F, Sasaguri T (2007). The Wnt/*β*-catenin signaling pathway as a target in drug discovery. *Journal of Pharmacological Sciences*.

[51] Ikeda S, Kishida M, Matsuura Y, Usui H, Kikuchi A (2000). GSK-3*β*-dependent phosphorylation of adenomatous polyposis cop gene product can be modulated by *β*-catenin and protein phosphatase 2A complexed with Axin. *Oncogene*.

[52] Lepourcelet M, Chen YNP, France DS (2004). Small-molecule antagonists of the oncogenic Tcf/*β*-catenin protein complex. *Cancer Cell*.

[53] Wang W, Liu H, Wang S, Hao X, Li L (2011). A diterpenoid derivative 15-oxospiramilactone inhibits Wnt/*Β*-catenin signaling and colon cancer cell tumorigenesis. *Cell Research*.

[54] Emami KH, Nguyen C, Ma H (2004). A small molecule inhibitor of *β*-catenin/cyclic AMP response element-binding protein transcription. *Proceedings of the National Academy of Sciences of the United States of America*.

[55] Chen B, Dodge ME, Tang W (2009). Small molecule-mediated disruption of Wnt-dependent signaling in tissue regeneration and cancer. *Nature Chemical Biology*.

[56] Shan J, Shi DL, Wang J, Zheng J (2005). Identification of a specific inhibitor of the Dishevelled PDZ domain. *Biochemistry*.

[57] Mita AC, Mita MM, Nawrocki ST, Giles FJ (2008). Survivin: key regulator of mitosis and apoptosis and novel target for cancer therapeutics. *Clinical Cancer Research*.

[58] Olmeda D, Castel S, Vilaró S, Cano A (2003). *β*-catenin regulation during the cell cycle: implications in G2/M and apoptosis. *Molecular Biology of the Cell*.

[59] Al-Husein B, Abdalla M, Trepte M, Deremer DL, Somanath PR (2012). Antiangiogenic therapy for cancer: an update. *Pharmacotherapy*.

[60] Rini BI, Atkins MB (2009). Resistance to targeted therapy in renal-cell carcinoma. *The Lancet Oncology*.

[61] Zhang L, Bhasin M, Schor-Bardach R (2011). Resistance of renal cell carcinoma to sorafenib is mediated by potentially reversible gene expression. *PLoS ONE*.

[62] Bhatt RS, Wang X, Zhang L (2010). Renal cancer resistance to antiangiogenic therapy is delayed by restoration of angiostatic signaling. *Molecular Cancer Therapeutics*.

[63] Cho IC, Chung J (2012). Current status of targeted therapy for advanced renal cell carcinoma. *Korean Journal of Urology*.

[64] Banumathy G, Cairns P (2010). Signaling pathways in renal cell carcinoma. *Cancer Biology and Therapy*.

[65] Reagan-Shaw S, Nihal M, Ahmad N (2008). Dose translation from animal to human studies revisited. *FASEB Journal*.

