# Supplementary material for: Ovatodiolide Targets β-Catenin Signaling in Suppressing Tumorigenesis and Overcoming Drug Resistance in Renal Cell Carcinoma
Source: Evid Based Complement Alternat Med. 2013 May 26;2013:161628. doi: 10.1155/2013/161628 (PMC3677612; doi:10.1155/2013/161628)
Supplement: Supplementary file 1 — Ovatodiolide specifically inhibits WNT/βcatenin signaling and therefore reduces TOP/FOP ratio (see Figure S1A-B), βcatenin activation and downstream gene expression (see Figure S1C-D). Its purity was also examined (see Figure S1E). Ovatodiolide inhibits RCC cell viability and induces apoptosis were confirmed in all four RCC cells and normal HK-2 cell lines (see Figure S1A-D). And sub-IC50 dose also resulted in similarly inhibitory effects (see Figure S1E). Ovatodiolide inhibitory abilities on cell migration, invasion and tumorigenicity were also confirmed in all four RCC cells (see Figure S3A-B). Ovatodiolide reduces βcatenin stability and activation on a 26S proteasome dependent manner (see Figure S3C-F). Ovatodiolide significantly suppresses in vivo tumorigenicity without recognizably systemic toxicity (see Figure S4A-D). AKT inhibitor treatment induced effects similar to that of ovatodiolide and constitutively active AKT abrogated the ovatodiolide-induced inhibition of WNT/β-catenin signaling (see Figure S5A-C and S6A). According to molecular docking simulation, ovatodiolide inserted into β-catenin enclosing Ser-552 residue, the AKT phosphorylation site, and may result in a stereochemical change to reduce its activation. [file 161628.f1.pdf]

Supplemental figure legends

Figure S1. Ovatodiolide specifically inhibits WNT/β-catenin signaling. (A) After 24 hr of transfection with TOPflash or FOPflash plasmids, each compound (20 and 40 μM) or DMSO was added to cells and lucifierase activity was measured after 24-hr treatment. The pGL4.71 renilla luciferase vector (Promega) was cotransfected as a transfection control, and luciferase activity was measured within 20 min. Psoralen was used as a WNT/β-catenin signaling inductive control. (B) After 24 hr of transfection, cells were treated with 25 ng/ml recombinant human WNT3a (rhWNT3a) or 20 mM LiCl containing ovatodiolide or DMSO for an additional 24 hr. The control group was ovatodiolide or DMSO alone. (C) Immunocytochemistry with 40 μM ovatodiolide shows inhibited β-catenin nuclear translocation and its downstream genes cyclin D1 and survivin. The positivity of each marker was quantified with Aperio ImageScope and Spectrum Software. Data are mean±SD of triplicate experiments. (D) Western blot assay of effect of 40 μM ovatodiolide on protein levels of active β-catenin (p-β-catenin [S552]) and its downstream genes (c-myc, cyclin D1 and survivin) and other WNT molecules (TCF4, LRP5/6, p-LRP5/6, Axin1 and dishevelled) in RCC cell lines ACHN and A498, human embryonic kidney HEK293T cells and normal kidney epithelial HK-2 cells. (E) Representative high-performance liquid chromatography (HPLC) of ovatodiolide revealed the purity of compounds to be ~95% pure.

Figure S2. As Figure 2, ovatodiolide inhibits RCC cell viability and induces apoptosis in ACHN and A498 RCC cell lines. (A) The effect of ovatodiolide on cell viability was evaluated in ACHN and A498 cells by MTT assay at various times. The IC_{50} at 48 hr and the relative viability (the percentage of MTT absorbance) were shown. Data are mean±SD of triplicate experiments. (B) The original data for Figure 2A and S2A were re-plotted with absorbance 570 nm on MTT assay by treatment time for RCC and

HK-2 cells. (C) Flow cytometry of the effect of ovatodiolide on cell cycle distribution. ACHN and A498 cells were treated with 40 μM ovatodiolide for 48 hr, and sub-G1 and G2/M populations were measured. Data are mean±SD of triplicate experiments. (D) Western blot analysis of protein levels of cleaved caspase 3, 8, 9, and PARP and apoptotic proteins Bax, Bid and PUMA and antiapoptotic proteins Bcl-2, Bcl-xL and survivin in ACHN and A498 cellstreated with ovatodiolide for 24 or 48 hr. (E) A sub-IC50 concentration (15μM) was also examined in Caki-1 and 786-O cells for 24h and 48h. The sub-IC50 concentration also reduced levels of active β-catenin (p-Ser552-β-catenin) and its downstream genes (c-myc, cyclin D1 and survivin) but not other WNT molecules (TCF4), LRP5/6 and its active phosphorylated form, Axin1, and disheveled.

Figure S3. As Figure 3, ovatodiolide inhibits RCC cell migration, invasion and tumorigenicity. (A) Wound healing assay of four cell lines with ovatodiolide concentrations for 24 and 48 hr. The wound-repair area was imaged under the same field every 24 hr and quantified. (B) Western blot analysis of protein levels of matrix metalloproteinase 2 (MMP-2) and MMP-9 with 20 μg of each cell lysate. Activities were evaluated by 0.1% gelatin zymography with 50 μg conditioned media. (C) RT-PCR quantification of effect of ovatodiolide (40 μM for 24 hr) on mRNA level of β-catenin. (D) Ovatodiolide for 24 hr dose-dependently reduced β-catenin nuclear transloacation in Caki-1 and 786-O cells. (E) Ovatodiolide-induced β-catenin degradation is not lysosome-mediated. Ovatodiolide co-treated with lysomal inhibitors (10 and 20 μM chloroquine or 10 and 20 μM NH_4Cl) for 48 hr had no effect on β-catenin protein levels. (F) Immunoprecipitation and western blot analysis of protein

levels of β-catenin – TCF4 and β-catenin – E-cadherin interaction in four RCC cells and HEK293T cells.

Figure S4. (A) 786-O and ACHN cells were xenografted each in six mice. Xenografted mice were treated with 100 μg/kg ovatodiolide and DMSO control were shown. (B) Tumor, lung and liver weight, and (C) Body weight of 50 μg/kg, 100 μg/kg ovatodiolide treatment or DMSO control. Data are mean±SD of triplicate experiments. (D) Ovatodiolide- and DMSO-treated 786-O- or ACHN-xenografted mice showed no systemic toxic effects. Representative sections of major organs, including brain (cerebrum and cerebellum), heart, liver, spleen, lung, kidney, stomach, intestine, and urine bladder, underwent hematoxylin and eosin staining.

Figure S5. (A) AKT inhibitor treatment induced effects similar to that of ovatodiolide and constitutively active AKT abrogated the ovatodiolide-induced inhibition of WNT/β-catenin signaling. In the left column, RCC cells were treated with 40 μM ovatodiolide, or co-treatment with AKT inhibitor VIII (5 μM) and ovatodiolide for 24 hr, and levels of p-β-catenin (S552) and β-catenin were examined. In the right column, RCC cells were transfected with 2μg constitutively active AKT plasmid (addgene#14751) for 24 hr, then treated with 40 μM ovatodiolide for an additional 24 hr. Ovatodiolide obviously reduced p-β-catenin (S552) and slightly reduced β-catenin. Constitutively active AKT induced higher levels of p-β-catenin (S552), p-Foxo3a (T32), p-mTOR (S2448) and p-p70S6K (T389), and it also abrogated most of the ovatodiolide inhibited p-β-catenin (S552) level. Nevertheless, ovatodiolide treatment did not modify AKT-induced p-Foxo3a (T32), p-mTOR (S2448) and p-p70S6K (T389) levels. (B) RT-PCR quantification of mRNA levels of WNT/β-catenin signaling targets

Axin2, Nkd1 and Sp5 in RCC cells treated with ovatodiolide (40 μM) with/without AKT inhibitors VIII (5 μM) or 2μg constitutively active AKT plasmid. (C) Schematic diagram depicts the mechanism of inactivation of β-catenin by ovatodiolide in RCC cells.

Figure S6. (A) The effect of constitutively active Akt also partially rescued the ovatodiolide-induced cell death. (B) The physically binding between ovatodiolide and beta-catenin was simulated on the molecular docking website PATCHDOCK with the 3D structure files for ovatodiolide (PubChem: CID_6451060) and beta-catenin (PDB: 1QZ7). The ovatodiolide inserted into the β-catenin molecule enclosing by the AKT phosphorylation site, Ser-552 residue, and may result in a stereochemical change to reduce its activation. There is no proper 3D structure including N-terminus of β-catenin and it is uneasy to evaluate whether ovatodiolide also bound to the GSK3β targeting Ser33, Ser37 or Thr41 residues.

Figure S1

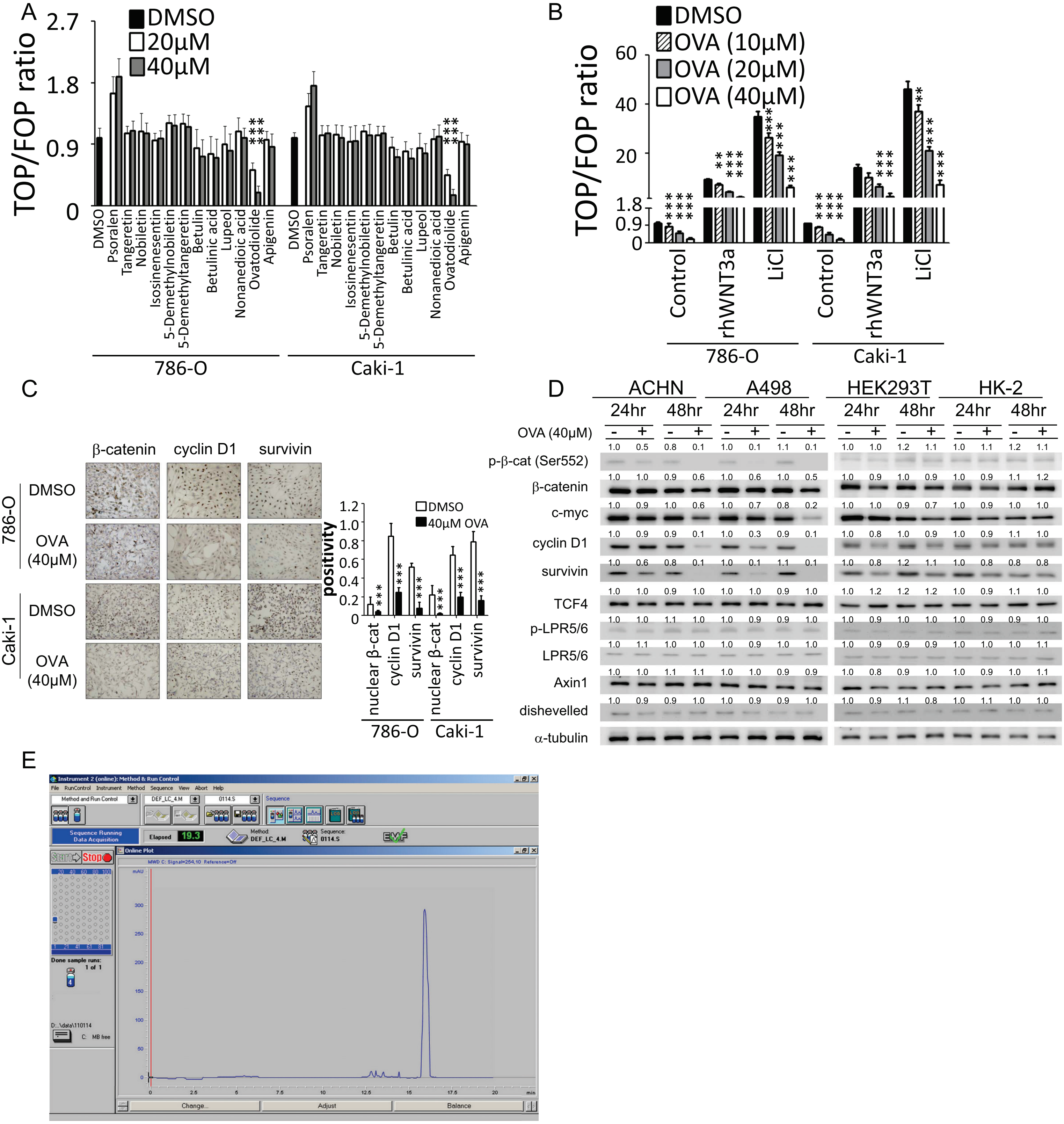
A
TOP/FOP ratio
2.7
1.8
0.9
0
DMSO
20μM
40μM
DMSO
Psoralen
Tangeretin
Nobiletin
Isosinenesentin
5-Demethylnobiletin
5-Demethyltangeretin
Betulin
Betulinic acid
Lupeol
Nonanedioic acid
Ovatodiolide
Apigenin
786-O
Caki-1
B
DMSO
OVA (10μM)
OVA (20μM)
OVA (40μM)
60
40
20
1.8
0.9
0
Control
rhWNT3a
LiCl
786-O
Caki-1
C
β-catenin
cyclin D1
survivin
786-O
DMSO
OVA (40μM)
Caki-1
positivity
1.2
1.0
0.8
0.6
0.4
0.2
0
DMSO
40μM OVA
nuclear β-cat
786-O
D
ACHN
A498
HEK293T
HK-2
24hr
48hr
OVA (40μM)
p-β-cat (Ser552)
c-myc
TCF4
p-LPR5/6
LPR5/6
Axin1
dishevelled
α-tubulin
E
Instrument 2 (online): Method & Run Control
Sequence Running
Data Acquisition
Elapsed
19.3
Online Plot
Done sample runs:
Change...
Adjust
Balance

Figure S2

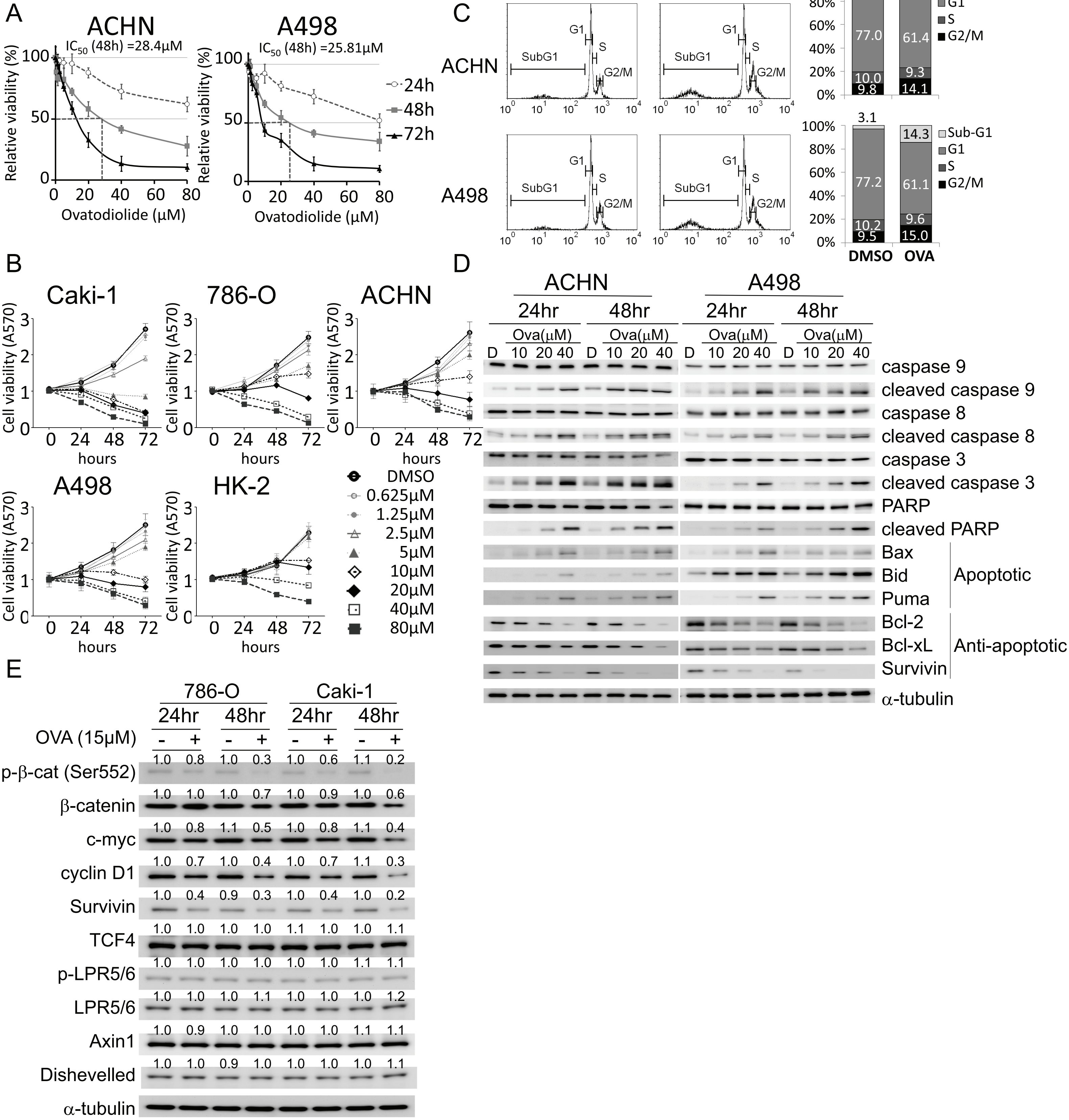
A
ACHN
IC50 (48h) =28.4μM
A498
IC50 (48h) =25.81μM
Relative viability (%)
Ovatodiolide (μM)
24h
48h
72h
B
Caki-1
786-O
ACHN
A498
HK-2
Cell viability (A570)
hours
DMSO
0.625μM
1.25μM
2.5μM
5μM
10μM
20μM
40μM
80μM
C
DMSO
OVA (40μM)
ACHN
A498
SubG1
G1
S
G2/M
Sub-G1
G1
S
G2/M
3.2
77.0
10.0
9.8
15.2
61.4
9.3
14.1
3.1
77.2
10.2
9.5
14.3
61.1
9.6
15.0
DMSO
OVA
D
ACHN
A498
24hr
48hr
Ova(μM)
D 10 20 40
caspase 9
cleaved caspase 9
caspase 8
cleaved caspase 8
caspase 3
cleaved caspase 3
PARP
cleaved PARP
Bax
Bid
Puma
Apoptotic
Bcl-2
Bcl-xL
Survivin
Anti-apoptotic
α-tubulin
E
786-O
Caki-1
24hr
48hr
OVA (15μM)
p-β-cat (Ser552)
β-catenin
c-myc
cyclin D1
Survivin
TCF4
p-LPR5/6
LPR5/6
Axin1
Dishevelled
α-tubulin

Figure S3

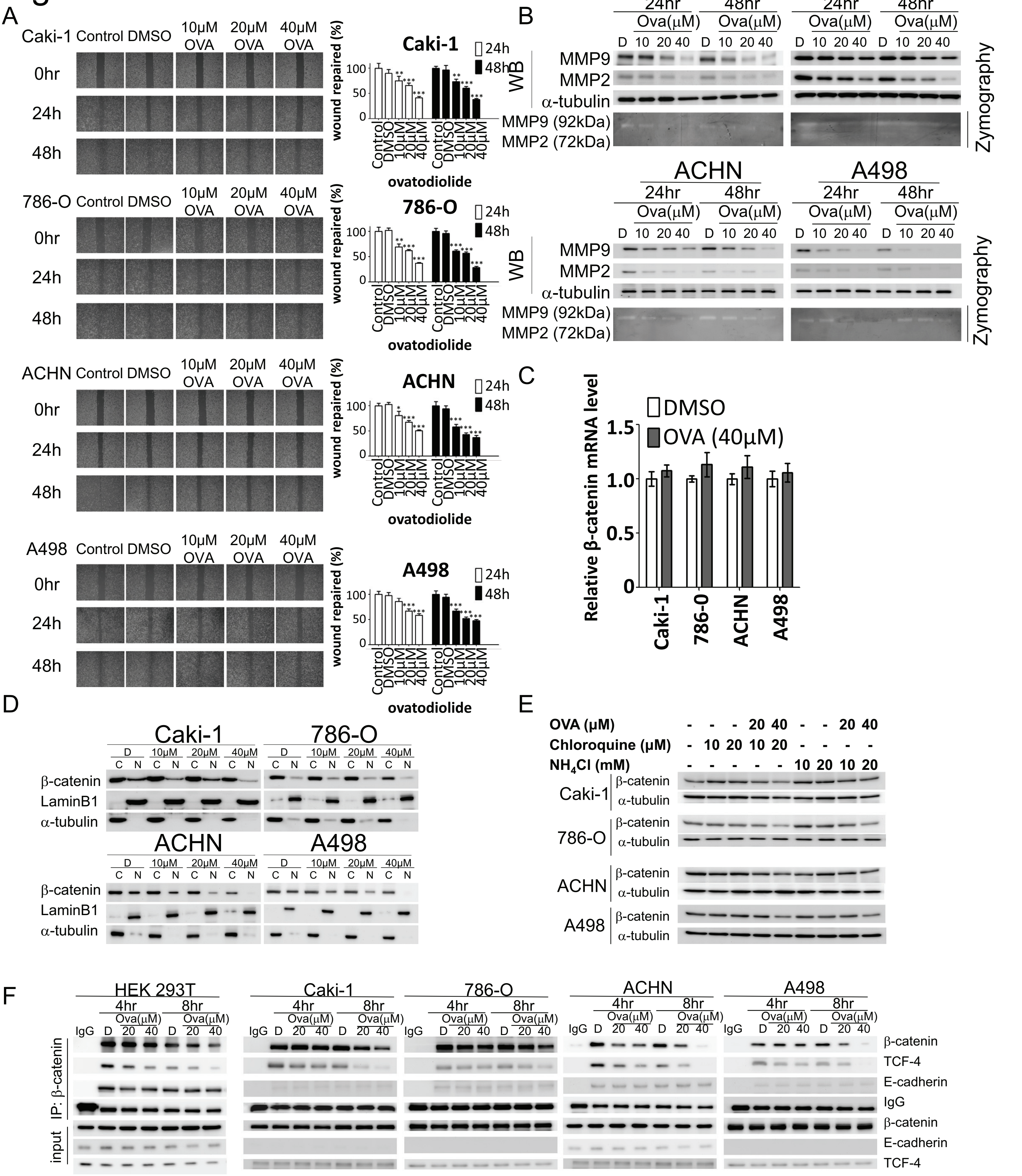
A
Caki-1 Control DMSO 10μM OVA 20μM OVA 40μM OVA
0hr
24h
48h
786-O
ACHN
A498
wound repaired (%)
24h
48h
ovatodiolide
Control DMSO 10μM 20μM 40μM
B
Caki-1
786-O
ACHN
A498
24hr
48hr
Ova(μM)
D 10 20 40
WB
MMP9
MMP2
α-tubulin
MMP9 (92kDa)
MMP2 (72kDa)
Zymography
C
Relative β-catenin mRNA level
DMSO
OVA (40μM)
D
β-catenin
LaminB1
α-tubulin
C N
E
OVA (μM)
Chloroquine (μM)
NH_4Cl (mM)
F
HEK 293T
IgG
4hr
8hr
IP: β-catenin
input
TCF-4
E-cadherin
IgG

Figure S4

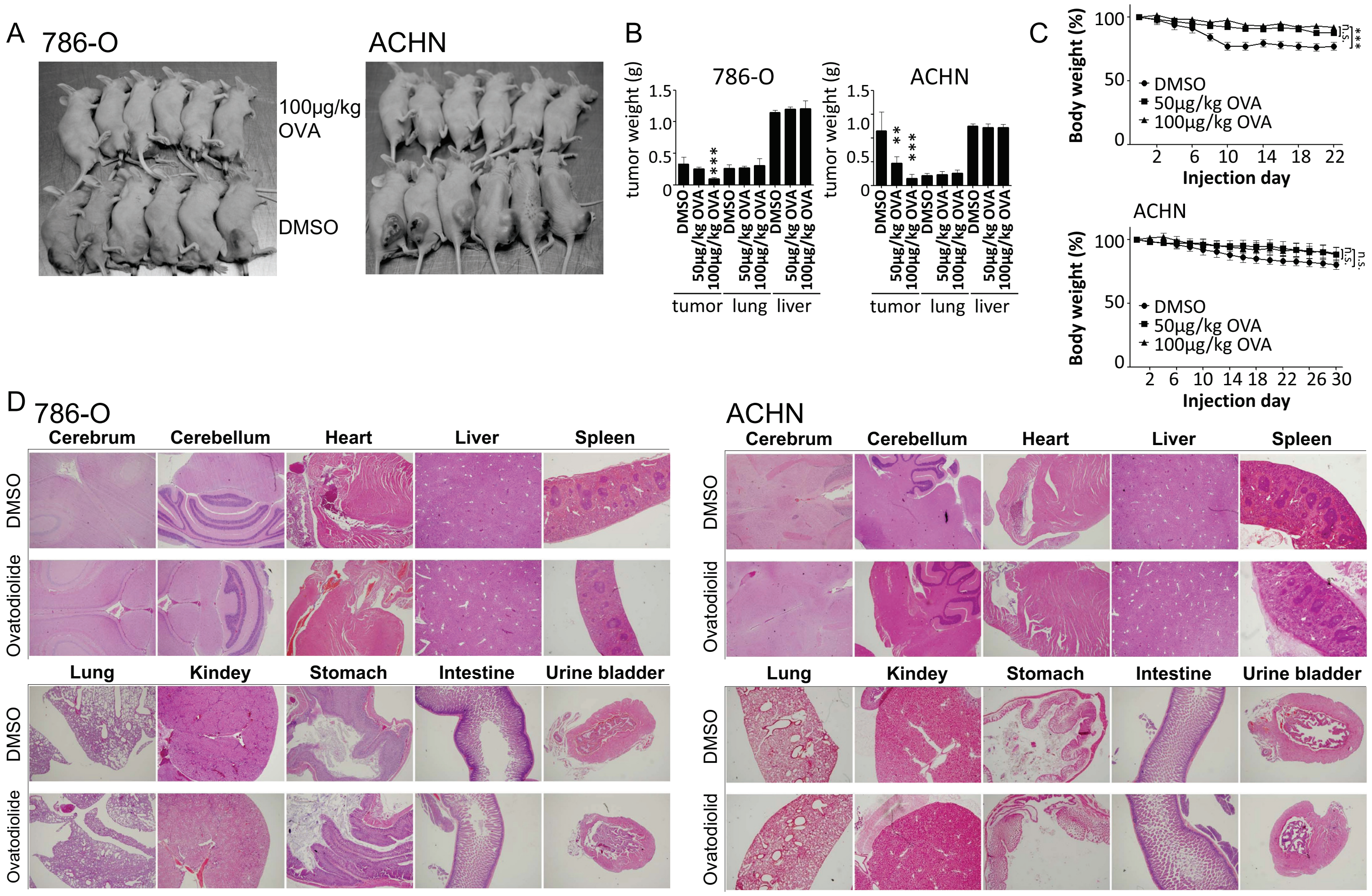
A
786-O
ACHN
100μg/kg OVA
DMSO
B
786-O
ACHN
tumor weight (g)
1.5
1.0
0.5
0
DMSO
50μg/kg OVA
100μg/kg OVA
tumor
lung
liver
C
786-O
ACHN
Body weight (%)
100
50
0
DMSO
50μg/kg OVA
100μg/kg OVA
Injection day
n.s.
D
786-O
ACHN
Cerebrum
Cerebellum
Heart
Liver
Spleen
DMSO
Ovatodiolide
Ovatodiolid
Lung
Kindey
Stomach
Intestine
Urine bladder

Figure S5

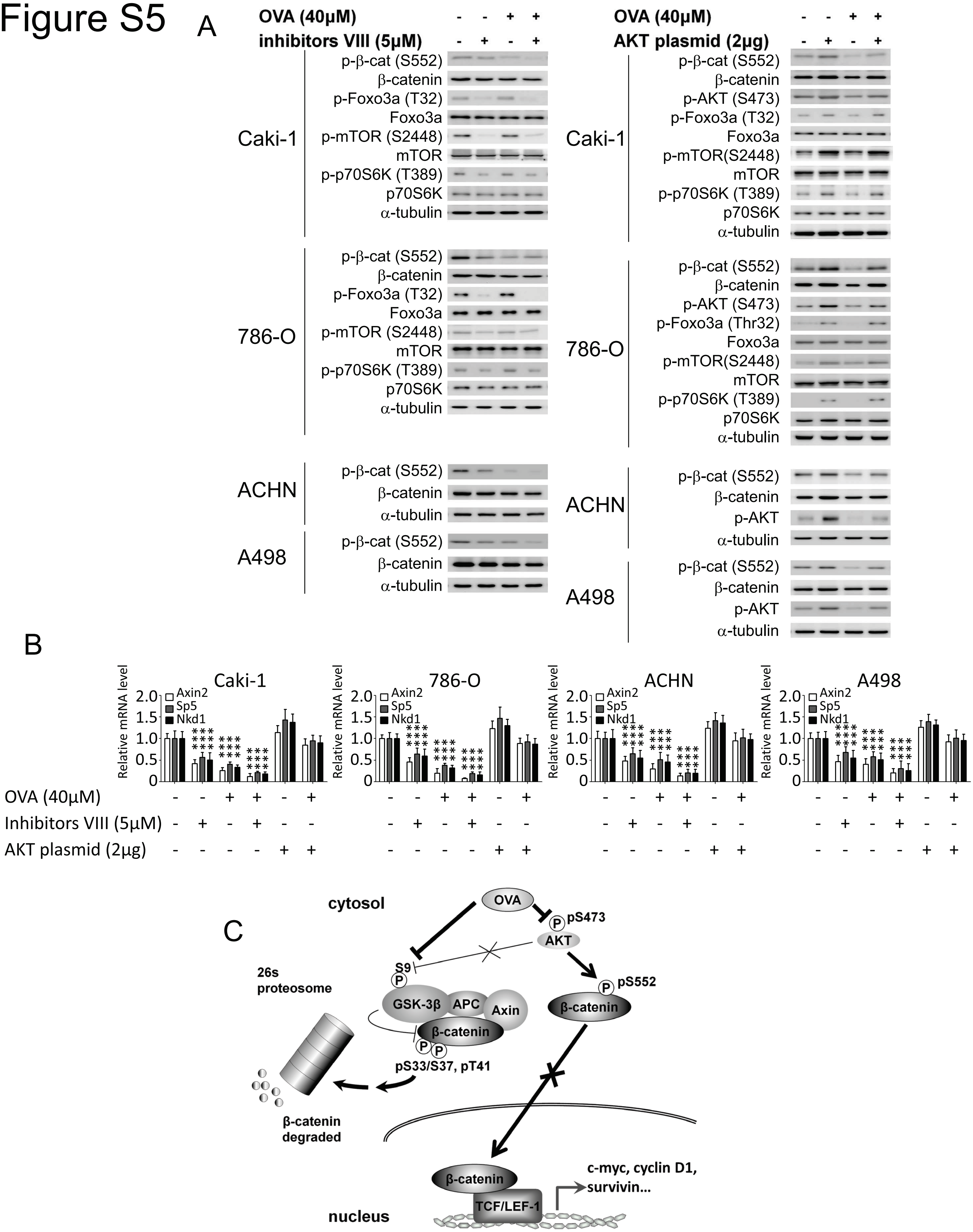
A
OVA (40μM)
inhibitors VIII (5μM)
AKT plasmid (2μg)
Caki-1
786-O
ACHN
A498
p-β-cat (S552)
β-catenin
p-Foxo3a (T32)
Foxo3a
p-mTOR (S2448)
mTOR
p-p70S6K (T389)
p70S6K
α-tubulin
p-AKT (S473)
p-Foxo3a (Thr32)
p-AKT
B
Relative mRNA level
Axin2
Sp5
Nkd1
OVA (40μM)
Inhibitors VIII (5μM)
AKT plasmid (2μg)
C
cytosol
nucleus
OVA
AKT
pS473
S9
26s proteosome
GSK-3β
APC
Axin
β-catenin
pS552
pS33/S37, pT41
β-catenin degraded
TCF/LEF-1
c-myc, cyclin D1, survivin...

Figure S6

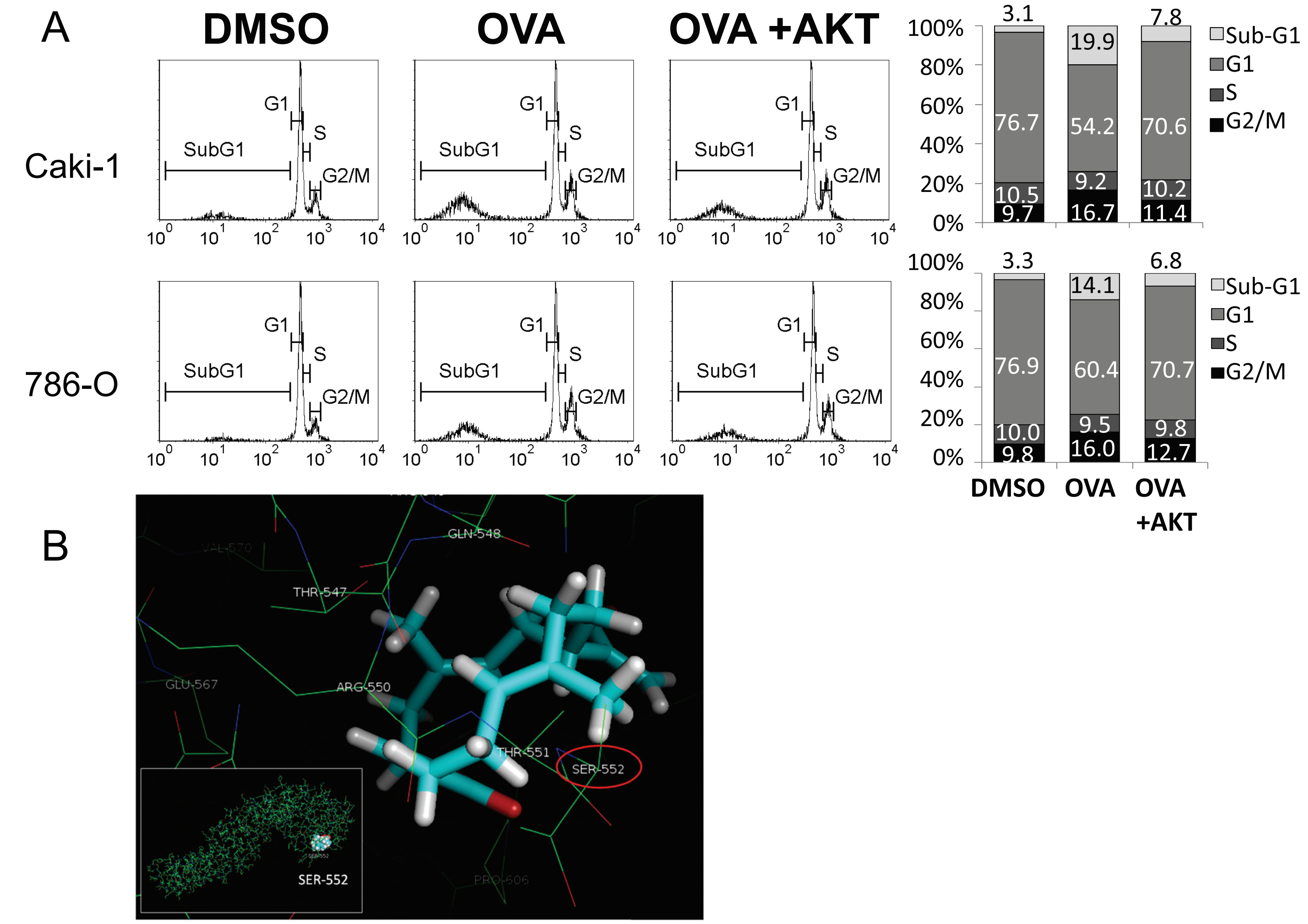

A
DMSO
OVA
OVA +AKT
Caki-1
786-O
SubG1
G1
S
G2/M
100%
80%
60%
40%
20%
0%
Sub-G1
G1
S
G2/M
3.1
76.7
10.5
9.7
19.9
54.2
9.2
16.7
7.8
70.6
10.2
11.4
3.3
76.9
10.0
9.8
14.1
60.4
9.5
16.0
6.8
70.7
9.8
12.7
DMSO
OVA
OVA +AKT
B
GLN-548
VAL-570
THR-547
GLU-567
ARG-550
THR-551
SER-552
PRO-606
SER-552

Table S1

Compound	PubChem BioActivity	
	Outcome	IC50
***Citrus reticulata* Blanco**		
Nobiletin	Active	40μM In vitro cytotoxic potency against NCI-60 human tumor cell line
Tangeretin	Active	41.2μM Growth inhibition of human SHSY5Y cells assessed as viable cells after 48 hrs by Trypan blue dye
5-Demethylnobiletin	Active	2.07μM Antiproliferative activity against HL60 after 24 hrs
5-Demethyltangeretin	Active	<= 50uM or explicitly reported as active by ChEMBL
Isosinensetin	Active	28μM Anticancer activity against human HT-29 cells after 72 hrs by MTT assay
β-Myrcene	Inactive	Not shown
α-Pinene	Inactive	Not shown
Naringin	Inactive	Not shown
Hesperetin	Inactive	Not shown
Limonene	Inactive	Not shown
Sabinene	Inactive	Not shown
Linalool	Inactive	Not shown
Hesperidin	Unspecified	142μM Anticancer activity against human HT-29 cells after 72 hrs by MTT assay
3-Carene	Unspecified	NA
Sinensetin	Unspecified	67μM Anticancer activity against human HT-29 cells after 72 hrs by MTT assay
β-Pinene	Untested	Not shown
Terpinolene	Untested	Not shown
Limonene oxide	Untested	Not shown
Decanal	Untested	Not shown
α-Phellandrene	Untested	Not shown
Tetramethyl-O-isoscutellarein	Untested	Not shown
***Hibiscus syriacus* L.**		
Betulin	Active	1.4μM Cytotoxicity against human WI 38 cells after 48 hrs by WST-8 assay
Betulinic acid	Active	0.026μM Cytotoxicity against human HeLa cells after 72 hrs by MTT assay
Lupeol	Active	<= 50uM or explicitly reported as active by ChEMBL
Nonanedioic acid	Active	42.1μM Cytotoxicity against human MCF7 cells by MTT assay
Suberic acid	Inactive	Not shown
Myristic acid	Inactive	Not shown
Palmitic aicd	Inactive	Not shown
Lauric acid	Inactive	Not shown
Canthin-6-one	Inactive	50μM Cytotoxicity against human HepG2 cells upto 50 uM by neutral red assay
(+)-pinoresinol	Inactive	Not shown
Syringaresinol	Unspecified	Cytotoxicity against human KB cells
Feruloyltyramines	Unspecified	38μMc Growth inhibition of mouse B16 2F2 cells after 3 days
1,22-Docosanediol	Unspecified	100μM, cytotoxicity against human A549 cells after 72 hrs by MTT assay
1-Octacosanol	Untested	Not shown
Erythrotriol	Untested	Not shown
3′-Hydroxydaidzein	Untested	Not shown

Table S1 (continued)

***Anisomeles indica* L.**		
Ovatodiolide	Active	1.4μM, HepG2 cells after 48 hrs
Apigenin	Active	35μM, cytotoxicity against human H9 cells after 3 days
Arjunolic acid	Inactive	Not shown
Citral	Inactive	Not shown
Limonene	Inactive	Not shown
Methyl gallate	Inactive	Not shown
Eugenol	Inactive	Not shown
Malic acid	Inactive	Not shown
Stigmasterol	Unspecified	50μM, cytotoxicity against human HeLa cells by MTT assay
Hederagenin	Unspecified	Not shown
Acetoside	Unspecified	Not shown
Isoacetoside	Untested	Not shown
Calceolarioside A	Untested	Not shown
Compneoside	Untested	Not shown
3, 4-Dihydroxybenzoic acid	Untested	Not shown
Terniflorin	Untested	Not shown
Anisofolin A	Untested	Not shown
Iso-ovatodiolide	Untested	Not shown
Anisomelic acid	Untested	Not shown
Borneol	Untested	Not shown
α- Terpineol	Untested	Not shown
Azullene	Untested	Not shown
Caryophyllene	Untested	Not shown

Table S2

Gene	Forward	Reverse
β-catenin	5'-AAAATGGCAGTGCGTTTAG-3'	5'-TTTGAAGGCAGTCTGTCGTA-3'
AXIN2	5'-CCCAAGCCCCATAGTGCCCAAAG-3'	5'-CAGGGGAGGCATCGCAGGGTC- 3'
SP5	5'-GCGGCGAGGGGCAAGGGC-3'	5'- CGCCGAGGCATGGACACCCG-3'
NKD1	5'-TCACTCCAAGCCGGCCGCC-3'	5'-TCCCGGGTGCTTCGGCCTATG-3'
GAPDH	5'-TGTTGCCATCAATGACCCCTT-3'	5'-CTCCACGACGTACTCAGCG-3'

Table S3

Gene	Manufacturer and product number
AKT	Cell Signal Tech.#4691
Phospho-AKT (Ser473)	Cell Signal Tech.#4060
Bax	Cell Signal Tech.#5023
Bcl-2	Epitomics #1017
Bcl-xL	Epitomics #1018
β-catenin	Epitomics #1247
Phospho-β-catenin (S552)	Cell Signal Tech.#9566
Phospho-β-catenin (S33/37,T41)	Cell Signal Tech.#9561
Bid	Clone FL-195, Santa cruz#SC-11423
Caspase-3	Cell Signal Tech. #9662
Cleaved Caspase-3	Cell Signal Tech. #9664
Caspase-8	Cell Signal Tech. #4790
Cleaved Caspase-8	Cell Signal Tech.#9496
Caspase-9	Cell Signal Tech.#9502
Cleaved Caspase-9	Cell Signal Tech.#9501
c-myc	Clone 9E11, Santa Cruz Biotech.#sc-47694
cyclin D1	Clone DCS-6, Cell Signaling Tech. #2926
E-cadherin	Epitomics #1702
ERK-1	Epitomics #1172
Phospho-ERK1 (T202)	Epitomics #1481
GAPDH	Epitomics #2251
GSK3	Cell Signal Tech.#9315
Phospho-GSK3 (Ser9)	Cell Signal Tech.#9322
Ki-67	Dako Clone MIB-1 #M7240
Lamin B1	Epitomics #6581
MEK-1	Epitomics #1235
Phospho-MEK1 (S217/221)	Epitomics #T3735
MMP-2	Lab Vision #RB-1537
MMP-9	Lab Vision # RB-1539
PARP	Cell Signal Tech.#9542
Cleaved PARP	Cell Signal Tech.#5625
PUMA	Cell Signal Tech.#4976
RAF1	Cell Signal Tech.#9422
Phospho-RAF1 (S338)	Cell Signal Tech.#9427
RAS	Cell Signal Tech.#3339
STAT3	Epitomics #2281
Phospho-STAT3 (Y705)	Epitomics #2236
Survivin	Epitomics #2463
TCF4	Epitomics #2114
α-tubulin	Clone DM1A, Lab Vision#MS-581
